# Effect of psychotropic medications on suicide-related outcomes: a systematic review and meta-analysis of observational studies

**DOI:** 10.1016/j.eclinm.2026.103800

**Published:** 2026-02-21

**Authors:** Stefaniya Kozhevnikova, Christina Emilian, Giulio Scola, Zheng Chang, Denis Yukhnenko, Seena Fazel

**Affiliations:** a Department of Psychiatry, University of Oxford, Oxford, United Kingdom; b Department of Social Policy and Intervention, University of Oxford, Oxford, United Kingdom; c Department of Medical Epidemiology and Biostatistics, Karolinska Institutet, Stockholm, Sweden

**Keywords:** Suicide, Self-harm, Psychopharmacology, Pharmacoepidemiology, Antidepressants

## Abstract

**Background:**

Psychiatric disorders are associated with increased risk of suicide-related outcomes, and the impact of pharmacological treatments on these outcomes is uncertain. Although randomised controlled trials are the main approach to evaluate efficacy, they may not provide externally valid results for suicide prevention in psychiatric populations. Thus, we aimed to synthesise the evidence on the effect of psychotropic medications on suicide-related outcomes from observational studies.

**Methods:**

In this systematic review and meta-analysis, we systematically searched Ovid (MEDLINE, Embase, APA PsychArticles, AMED, BIOSIS, Global Health, PsycINFO), and Web of Science Core Collection from database inception to 8 December 2025 for pharmacoepidemiological and other observational studies on suicide-related outcomes in people treated with the main types of psychotropic medications: antidepressants, antipsychotics, mood stabilisers (including antiepileptics), and medications for anxiety (anxiolytics), attention deficit and hyperactivity disorder (ADHD), and substance use disorder (SUD). We included primary studies involving adults with common psychiatric diagnoses (schizophrenia spectrum disorders, bipolar disorder, depressive disorders, and personality disorders), who were prescribed medication and a comparison sample with the same diagnosis without prescribed medication (between-individual studies) or the same individuals during a non-prescription period (within-individual studies). We excluded studies that did not report psychiatric diagnoses and from selected samples. Outcomes were suicide attempts/self-harm and suicide mortality. We pooled effect sizes as odds ratios (OR), hazard ratios (HR) or risk ratios (RR) using random-effects models and assessed study quality using NOS and QUIPS tools. Study protocol was registered with PROSPERO, CRD42024515794.

**Findings:**

Of 5653 records identified, 48 independent studies from 13 countries based on more than 6 million people (47% male) met inclusion criteria. Across the main diagnostic categories and 70 individual medications examined, associations with reducing risk of suicide mortality were found for second generation antipsychotics in schizophrenia spectrum disorders: clozapine (OR = 0.40; 0.36–0.60; I^2^ = 60%, moderate certainty), olanzapine (OR = 0.53; 0.39–0.71; I^2^ = 34%, high certainty), quetiapine (OR = 0.75; 0.58–0.96; I^2^ = 0%, high certainty), and zuclopenthixol (OR = 0.44; 0.30–0.63; I^2^ = 0%, high certainty). In schizophrenia, second generation antipsychotics were also associated with reduced risks of suicide attempts: olanzapine (OR = 0.76; 0.60–0.98; I^2^ = 84%, moderate certainty) and risperidone (OR = 0.61; 0.52–0.72; I^2^ = 57%, moderate certainty). In bipolar disorder, lithium (OR = 0.38; 0.28–0.50; I^2^ = 67%, moderate certainty) and valproic acid (OR = 0.66; 0.59–0.75; I^2^ = 0%, high certainty) were associated with lower suicide risks, and lithium was also associated with lower risks of suicide attempts (OR = 0.60; 0.44–0.82; I^2^ = 92%, moderate certainty). In depression, associations with lower risk of suicide mortality for selective serotonin reuptake inhibitors (SSRIs) (OR = 0.61; 0.47–0.81; I^2^ = 23%, high certainty) and tricyclic antidepressants (OR = 0.68; 0.59–0.78; I^2^ = 0%, high certainty) were found. Benzodiazepines were associated with higher risk of suicide mortality in most diagnostic categories, except depression. There was some evidence for publication bias for lithium in bipolar disorder and clozapine in schizophrenia spectrum disorders, leading to more papers reporting lower risks of suicide-related outcomes. The risk of bias in included studies was low in 47 studies, moderate in one study, and certainty of evidence was moderate.

**Interpretation:**

There is evidence of varying effects of psychotropic medication on the risk of suicide-related outcomes across different psychiatric disorders. The appropriate use of prescribed medications in people with high risks of suicide-related outcomes is an important suicide prevention strategy. Findings are not causal, and limitations include the observational nature of included studies, risk of residual confounding, high heterogeneity for some outcomes, and moderate quality of the evidence.

**Funding:**

10.13039/501100000769University of Oxford (Hill Foundation), NIHR Oxford Health Biomedical Research Centre, 10.13039/100010269Wellcome Trust.

**Research in context**

**Evidence before this study**

We searched published literature (using PubMed, Embase, PsycINFO, Medline, and Google Scholar) for systematic reviews of psychopharmacological treatments for suicide-related outcomes in the general population published in English from inception to 8 December 2025, using the following search terms: “(systematic review or meta-analysis or meta-review or umbrella review) and (self-harm or self harm or selfharm or suicid∗) and (antidepressant∗ or SSRI∗ or TCA∗ or SNRI∗ or NASSA∗ or SARI∗ or MAO∗ or anti-anxiety or tranquil∗ or benzodiazepin∗ or anxiolytic∗ or anxiolitic∗ or antipsychotic∗ or neuroleptic∗ or mood stabili∗ or antiepileptic∗ or lithium)”. Identified reviews were either conducted on specific population groups, specific medication classes, specific diagnostic categories or synthesised data from randomised controlled trials. One review with broader inclusion criteria (i.e. different populations, diagnostic categories, and study designs) limited observational studies to within-group analysis, and pooled trial and observational data together. Lithium and clozapine consistently showed a strong association with reduced risks of suicide-related outcomes, while findings for antidepressants and other antipsychotics varied between different population groups and diagnoses.

**Added value of this study**

This meta-analysis synthesised data from over 30 years of observational studies on the effects of 70 different medications on suicide attempts/self-harm and suicide in over 6 million people. While study findings are consistent with previous research on lithium and clozapine, we found diagnosis-specific evidence of medications being associated with lower risk of suicidal behaviour, particularly for some second-generation antipsychotics in schizophrenia spectrum disorders and two classes (SSRIs and tricyclics) of antidepressants in depression.

**Implications of all the available evidence**

Certain psychotropic medications are associated with reducing risk of suicide-related outcomes in people with psychiatric disorders. These data suggest that personalising medication for specific diagnoses may have a role in suicide prevention in high-risk psychiatric populations. The results are not causal and may be influenced by indication bias. Future studies should report outcomes separately by patient diagnosis, and further within-individual and trial target emulation studies are needed.

## Introduction

Epidemiological studies have consistently shown that psychiatric disorders are strongly associated with an increased risk of suicidal behaviour. In depression, population-based studies have reported increased risk of suicide attempts during acute episodes and in partial remission.^1^ In schizophrenia^2^ and bipolar disorder,^3^ relative risks are more than 10-fold higher for suicide attempts and mortality compared with the general population, confirmed when using non-affected sibling comparisons.^2^^,^^3^ Psychological autopsy investigations are consistent with these increased risks.^4^ Consequently, suicide prevention strategies are targeted to these high-risk populations, which include pharmacological, psychological and social interventions.

Although trial evidence finds that certain psychological treatments can prevent self-harm, and reducing access to means has strong and consistent observational evidence in support,^5^ the evidence for the efficacy of medication in preventing suicide-related outcomes (suicide attempts/self-harm and suicide mortality) has been inconsistent. A recent synthesis of trial evidence^6^ found small increased risks of self-harm and suicide in the community for some selective serotonin reuptake inhibitors (SSRI) antidepressants and antipsychotics, but risks were moderately reduced for specific medications, such as the SSRI citalopram and antipsychotic paliperidone, and lithium reduced the risk of suicide mortality. In this previous review, other medications had uncertain findings due to the low number of participants recruited to the trials. Moreover, due to selection criteria for participation, randomised controlled trials (RCTs) do not recruit people at risk of suicidal behaviours and hence their results have limited generalisability to clinical populations.^7^ This may lead to the lower external validity of the RCT findings in relation to suicide, as the recruited trial sample and real-world treated population can markedly differ. While controlled trials measure the intended effect of an intervention in ideal circumstances (efficacy), observational studies collect real-world data from clinical practice (effectiveness).^8^^,^^9^

Previous reviews on the effect of medications on suicide-related outcomes have focused on specific population groups, medication classes, diagnostic categories or synthesised RCT data. One review with broader inclusion criteria^10^ limited observational studies to those that conducted within-group analyses, and combined trial and observational data. More generally, lithium and the antipsychotic clozapine have consistently showed a strong association with reduced risk of suicide-related outcomes,^6^^,^^10^, ^11^, ^12^, ^13^ while findings for antidepressants and other antipsychotics have varied between different population groups and diagnoses.^6^^,^^10^^,^^11^^,^^14^, ^15^, ^16^, ^17^

To address the limited evidence-base on the impact of some medications, observational data are increasingly examined as part of pharmacoepidemiological studies, which apply epidemiological methods on large populations^18^ to assess treatments. These studies can examine medication effectiveness and adverse effects, assess patterns of medication use, and allow for comparison of findings from clinical trials with those on medication effects in clinical practice.^19^ However, a key limitation of observational studies is the potential for confounding of the treatment effect on the outcome. Approaches to address this issue depend on the study design. In between-individual studies, rates of suicide-related outcomes are compared between individuals prescribed medications and those who are not. In these studies, depending on the research question, baseline and occasionally time-varying confounding need addressing. Different methods can be used to account for measured baseline confounders, such as conditioning on them (regression adjustment)^20^ and propensity score methods.^21^ For measured time-varying confounders affected by past treatment, advanced statistical methods are required, specifically g-methods.^22^ Despite accounting for measured confounders, residual confounding cannot be entirely ruled out. In within-individual studies (also known as self-controlled or case-crossover studies), suicide rates in participants during the treatment period are compared to suicide rates during periods without treatment.^23^ This design inherently accounts for all time-invariant confounders (e.g. genetic risks and childhood environment). However, time-varying confounding is not typically addressed and selection bias may occur, as included individuals in those studies need to have periods on and off medication. In addition, findings rely on length of the follow-up period.^24^

Despite various sources of confounding in observational studies, they provide useful real-world evidence of medication effectiveness in clinical practice, and do not have some of the limitations inherent to trial data, such as samples that exclude some high-risk patient populations (e.g. those with a history of self-harm, expressing suicidal thoughts at the time of trial recruitment, or who have other comorbidities) and typically small numbers of participants. Synthesising data from multiple high-quality observational studies with different methodological approaches (between- and with-individual epidemiological designs) can limit the risk of bias. This review's purpose is to synthesise the observational data on associations between psychopharmacological treatments and suicide-related outcomes by different psychiatric diagnoses, and to consider this real-world observational evidence in light of existing trial findings.

## Methods

### Search strategy and selection criteria

We followed the Preferred Reporting Items for Systematic Reviews and Meta-analyses guidelines (PRISMA).^25^ We searched several databases: Ovid (MEDLINE, Embase, APA PsychArticles, AMED, BIOSIS, Global Health, PsycINFO), and Web of Science Core Collection. No publication date or language restrictions were set. The same keywords were used for all databases for medications and study designs (see Supplementary Materials S1). We used individual names of medications from Stahl's Prescriber's guideline^26^ and broad medication classes (i.e. antipsychotics, antidepressants, anxiolytics, antiepileptics, and ADHD medications). We excluded some newer medications from our search, including ketamine or psilocybin, due to their role in treatment-resistant and specific conditions, such as cancer, which may create a selection bias and lower comparability to more conventional treatment studies. The search was updated on 28 March 2025 with a further update on 8 December 2025 (as part of the peer review process). The protocol was registered with Prospero (ID CRD42024515794) and amended with study changes.

Outcomes included the following for suicide-related outcomes: suicide mortality and suicide attempt/self-harms covered by ICD-10 codes X60-84 and Y10-34, and their equivalents in ICD-8/9.^27^ We excluded suicidal ideation (which is typically measured by psychometric tests such as the Columbia-Suicide Severity Rating Scale), which rely on self-reported data. However, the focus of this review was self-harm and suicide, which can be more reliably collected from healthcare and mortality registries than ideation from self-reported data, and aligns with national public health and health service priorities.

We included studies with the following designs: prospective and retrospective cohort studies (including effectiveness studies), case–control, and case-crossover studies. We excluded cross-sectional designs and case reports. Our focus was to identify studies investigating the effects of medication treatment on suicide-related outcomes. The medications examined were: antidepressants, anti-anxiety medications (anxiolytics), mood stabilisers (including antiepileptics), antipsychotics, and attention deficit and hyperactivity disorder (ADHD) medications. We excluded papers focused on suicide-related outcomes as a side effect (i.e. after hours or days), since suicide-related outcomes as adverse events are rare,^28^ and unlikely to be reported in observational studies (which rely on healthcare registers where symptoms are typically not routinely reported) and the aim was to investigate medium and longer term effects. Inclusion of shorter follow-up periods could lead to further confounding, where potential suicidal-related outcomes were related to the underlying condition rather than medication. We did not exclude non-suicidal self-harm from the suicide attempts category due to diagnostic challenges in separating it from suicide attempts.^29^ Instead, we included all self-harm acts as one of the outcomes (and suicide mortality the other). We excluded studies conducted exclusively in one age group (such as children), and in selected populations (e.g. people in prison, inpatient-only samples, those who are physically ill). Diagnoses without at least two studies investigating medications were not included in our review. If a study was conducted in two different populations with different diagnoses, the data from such study were separately synthesised in both relevant categories.

The first author, SK, conducted abstract and full-text screening, and a doctoral student CE undertook screening of random 20% of both screenings independently. The inter-rater reliability (Cohen's Kappa) between reviewers was 0.96. SK extracted the data using the standardised form, which included study and population characteristics and the statistic used to estimate the medication effects. CE independently verified extracted data and both extractors then met to compare their findings. Quality assessment of the studies was conducted independently by SK and CE using the Newcastle-Ottawa Scale for observational studies^30^ and the Quality In Prognosis Studies (QUIPS) tool.^31^ All disagreements between screening and assessment decisions were resolved in discussion with supervisors, SF and DY.

We conducted a “leave-one-out” sensitivity and the funnel plot publication bias analyses for the results where the number of medication studies were ≥5. Since statistical testing for publication bias is recommended to be only conducted in the analyses with ten or more included studies,^32^ we did not further investigate potential publication bias. We applied the Grading of Recommendations Assessment, Development and Evaluation (GRADE)^33^^,^^34^ framework to assess the certainty of evidence for the findings.

### Data analysis

We used a random effects meta-analysis to calculate odds ratios for all suicide behaviours and suicide mortality for different medications investigated in two or more studies. We synthesised the data for all medications separately, due to trial data previously reporting different effects of individual medications within one medication class^6^; if only medication class was mentioned in the included studies, we analysed those separately and referred to them as unspecified. For missing raw data, we used the Wald-type confidence intervals to convert reported effect sizes into a common effect size.^35^ Due to the rarity of outcomes investigated, we assumed that hazard ratios, odds ratios and risk ratios could be considered as broadly equivalent,^36^ which is statistically appropriate for the outcomes that occur in less than 10% of study population.^37^^,^^38^ In some cases, separate comparisons from one study had to be included in one model (i.e. paper studied different methods of assisting medication separately). In such cases, a multilevel random effects model was used, where the study ID was used as an underlying layer so that in the final model the data from one study were evaluated as such, avoiding calculating controls multiple times. Data analysis was conducted using the ‘Metafor’ and data were visualised using the ‘ggplot2’ packages in R version 4.3.1.

Serotonin-norepinephrine reuptake inhibitor (SNRI) antidepressants (venlafaxine, nefazodone), and noradrenergic and specific serotonergic antidepressants (NaSSA; mirtazapine) were analysed as one category because they were reported together in one included investigation.^39^ The data for both outcomes visualised together for better interpretability.

### Role of the funding source

The funder of the study had no role in study design, data collection, data analysis, data interpretation, or writing of the report. All authors had full access to all the data in the study and accept responsibility to submit for publication.

## Results

**Fig. 1:**
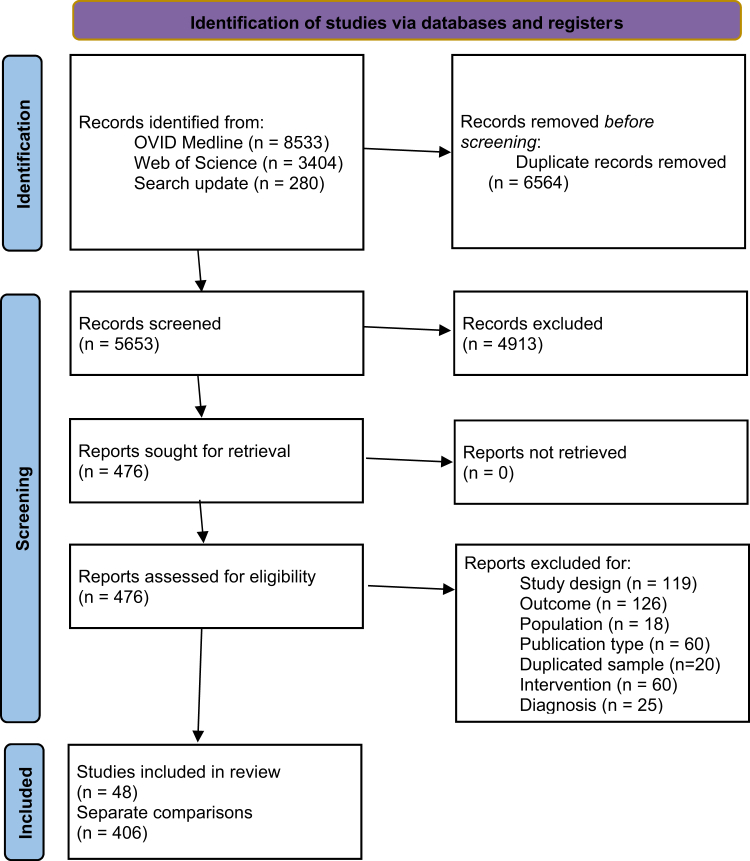
PRISMA flowchart.

All included studies are listed in Table 1, and the characteristics of included studies are presented in Table 2. Overall, 48 studies were included with 406 comparisons of medication with no medication (Fig. 1). The total number of participants was 6,489,573 people (estimated mean age 41.6 years; estimated 46.6% male). The full results of all pooled results with the heterogeneity I^2^ are available in Supplementary Materials S2. The full list of studies excluded on a full-text screening stage is available in Supplementary Materials S3.

**Table 1:** All included studies.

First author, year	Country	Study design	Diagnoses	Medications included	Outcomes studied	Risk of bias
Antolin-Concha, 2020^40^	Finland	WI	Bipolar disorder	Antidepressants: unspecified, amitriptyline, citalopram, doxepin, duloxetine, escitalopram, fluoxetine, mianserin, mirtazapine, moclobemide, paroxetine, sertraline, venlafaxine Antipsychotics: unspecified Antiepileptics: Carbamazepine, gabapentin, lamotrigine, oxcarbazepine, topiramate, valproic acid Anxiolytics: unspecified Mood stabilisers: unspecified, lithium	Self-harm repetition	QUIPS—low NOS—9
Arana, 2010^41^	UK	BI	Bipolar disorder, depression, epilepsy	Antiepileptics (unspecified)	Suicide attempt	QUIPS—low NOS—9
Asp, 2021^42^	Sweden	BI	Depression	Antipsychotics (unspecified)	Suicide attempt	QUIPS—low NOS—8
Cato, 2019^43^	Sweden	BI	Anxiety, bipolar disorder, depression, personality disorder, schizophrenia, SUD, ADHD	Antiepileptics (unspecified) Antipsychotics (unspecified) Antidepressants (unspecified), benzodiazepines, lithium, psychostimulants	Suicide death	QUIPS—low NOS—8
Chen, 2023^44^	Taiwan	BI	Bipolar disorder	Mood stabilisers: unspecified, lithium, carbamazepine, lamotrigine, valproic acid, zotepine	Suicide death	QUIPS—low NOS—9
Chen, 2024^45^	Taiwan	BI	Schizophrenia	Antipsychotics: Amisulpride, aripiprazole, chlorpromazine, clotiapine, clozapine, flupentixol, haloperidol, olanzapine, paliperidone, quetiapine, risperidone, zotepine	Suicide death	QUIPS—low NOS—9
Ekinci, 2022^46^	Turkey	BI	Schizophrenia	Benzodiazepines	Suicide attempt	QUIPS—low NOS—9
Fitzgerald, 2022^47^	Denmark	BI, WI	Bipolar disorder	Antipsychotics: unspecified Mood stabilisers: unspecified, lithium, valproate	Self-harm, suicide death	QUIPS—low NOS—9
Gibbons, 2009^48^	USA	BI, WI	Bipolar disorder	Antiepileptics: unspecified, carbamazepine, divalproex, gabapentin, lamotrigine, lithium, oxcarbazepine, topiramate	Suicide attempt	QUIPS—low NOS—9
Gibbons, 2010^49^	USA	WI	Bipolar disorder, depression, epilepsy, schizophrenia	Antiepileptics: Gabapentin	Suicide attempt repetition	QUIPS—low NOS—9
Haukka, 2008^50^	Finland	BI	Schizophrenia	Antidepressants: unspecified, citalopram, fluoxetine, mianserin, paroxetine, sertraline Antipsychotics: unspecified, clozapine, haloperidol, olanzapine, perphenazine, zuclopenthixol	Suicide attempt, suicide death	QUIPS—low NOS—9
Hayes, 2022^51^	UK	WI	Personality disorders	Antipsychotics: Quetiapine	Self-injury	QUIPS—low NOS—9
Herttua, 2022^52^	Denmark	Bi, WI	Personality disorders	Antipsychotics: unspecified, in men and women separately	Suicidal behaviour	QUIPS—low NOS—9
Isometsa, 2014^53^	Finland	BI	Bipolar disorder	Mood stabilisers: Lithium	Suicide death	QUIPS—low NOS—9
Kessing, 2005^54^	Denmark	BI	Bipolar disorder	Mood stabilisers: Lithium	Suicide death	
Kiviniemi, 2013^55^	Finland	BI	Schizophrenia	Antidepressants: unspecified, citalopram, fluoxetine, mirtazapine, venlafaxine Antipsychotics: unspecified, FGA, SGA, chlorprothiexine, clozapine, levomepromazine, olanzapine, perphenazine, quetiapine, risperidone, thioridazine	Suicide death	QUIPS—low NOS—9
Lagerberg, 2023^56^	Sweden	BI	Depression	Antidepressants: SSRIs	Suicide attempt	QUIPS—low NOS—9
Leon, 2014^57^	USA	WI	Bipolar disorder, depression	Antidepressants: (unspecified) Lithium Antipsychotics: SGA	Suicide death	QUIPS—low NOS—9
Lee, 2025^58^	South Korea	BI	Bipolar disorder, Schizophrenia	Clozapine, lithium, sodium valproate	Suicide death	QUIPS—low NOS—9
Lieslehto, 2023^59^	Sweden	BI, Wi	Borderline personality disorder	ADHD medications: unspecified, atomoxetine, dexamfetamine, lisdexamphetamine, methylphenidate Antidepressants: unspecified, agomelatine, amitriptyline, bupropion, citalopram, clomipramine, duloxetine, escitalopram, fluoxetine, fluvoxamine, mirtazapine, moclobemide, paroxetine, sertraline, venlafaxine, vortioxetine Antipsychotics: unspecified, long-acting injectables, aripiprazole, clozapine, flupentixol, haloperidol, levomepromazine, olanzapine, quetiapine, risperidone, zuclopenthixol Antiepileptics: Carbamazepine, lamotrigine, topiramate, valproic acid Mood stabilisers: unspecified, lithium	Suicide attempt	QUIPS—low NOS—9
Lin, 2023^60^	Taiwan	BI	Bipolar disorder	Mood stabilisers (unspecified)	Suicide death	QUIPS—low NOS—9
Ma, 2018^61^	Taiwan	Bi	Schizophrenia	Antipsychotics: Amisulpride, chlorpromazine, clothiapine, clozapine, flupentixol, haloperidol, olanzapine, quetiapine, risperidone, sulpiride, zotepine	Self-harm	QUIPS—low NOS—9
Marangell, 2008^62^	Multiple	BI	Bipolar disorder	Antidepressants: SSRIsAntiepileptics: Carbamazepine, oxcarbazepine, lamotrigine, lithium, valproate	Suicide attempt	
Montout, 2002^63^	France	BI	Schizophrenia	Antipsychotics: Benzamides, butyrophenoned, phenothiazines, thioxanthenes	Suicide death	QUIPS—low NOS—8
Neuner, 2011^64^	Germany	BI	Schizophrenia, affective disorders	Antiepileptics: unspecified, lithium Antipsychotics: FGA, SGA Antidepressants: SSRIs, MAOs, NaSSAs, SNRIs, TCAs; benzodiazepines	Suicide death	QUIPS—low NOS—8
Ng, 2023^65^	Hong Kong	WI	Bipolar disorder	Antiepileptics (unspecified)Antipsychotics (unspecified) Mood stabilisers: Lithium	Suicide attempt	QUIPS—low NOS—9
Olfson, 2006^66^	USA	BI	Depression	Antidepressants: unspecified, SSRIs, TCAs, bupropion, citalopram, fluoxetine, fluvoxamine, mirtazapine, nefazodone, paroxetine, sertraline, trazodone, venlafaxine	Suicide attempt, suicide death	QUIPS—low NOS—9
Olfson, 2008^67^	USA	BI	Depression	Antidepressants: unspecified, SSRIs	Suicide attempt	QUIPS—low NOS—9
Pfeiffer, 2013^68^	USA	BI	Depression	Benzodiazepines	Suicide death	QUIPS—low NOS—8
Reutfors, 2013^69^	Sweden	BI	Schizophrenia	Antidepressants: SSRIs Antipsychotics: Clozapine, risperidone, olanzapine, ziprasidone Mood stabilisers: Lithium	Suicide death	QUIPS—low NOS—8
Ringback Weitoft, 2014^70^	Sweden	BI	Schizophrenia	Antipsychotics: Aripiprazole, clozapine, flupenthixol, haloperidol, olanzapine, perphenazine, quetiapine, risperidone, ziprasidone, zuclopenthixol	Suicide attempt, suicide death	QUIPS—low NOS—9
Rohde, 2020^71^	Denmark	WI	Depression	ADHD medications: Methylphenidate, modafinil	Suicide attempt	QUIPS—low NOS—9
Rohde, 2021^72^	Denmark	WI	ADHD, personality disorders, schizophrenia	ADHD medications: Methylphenidate	Suicide attempt	QUIPS—risk of attrition NOS—7
Ruengorn, 2012^73^	Thailand	BI	Depression	Antipsychotics (unspecified) Benzodiazepines Antidepressants: SSRIs, TCAs	Suicide attempt	QUIPS—low NOS—8
Sondergard, 2007^39^	Denmark	BI	Depression	Antidepressants: unspecified, older antidepressants, SSRIs, new non-SSRIs (NaSSAs and SNRI)	Suicide death	QUIPS—low NOS—9
Sondergard, 2008^74^	Denmark	BI	Bipolar disorder	Antiepileptics: (unspecified), lithium	Suicide death	QUIPS—low NOS—9
Song, 2017^75^	Sweden	BI, WI	Bipolar disorder	Mood stabilisers: Lithium, valproate	Suicide attempt, repetition	QUIPS—low NOS—9
Suominen, 2009^76^	Finland	BI	Depression	Antidepressants: (unspecified), Antipsychotics: (unspecified)	Suicide death	QUIPS—low NOS—9
Taipale, 2020^77^	Finland	BI	Schizophrenia	Antipsychotics: Aripiprazole (oral), chlorprothiexine (oral), flupentixol (LAI & oral), fluphenazine (LAI), haloperidol (LAI & oral), levomepromazine (oral), olanzapine (LAI & oral), perphenazine (LAI & oral), quetiapine (oral), risperidone (LAI & oral), zuclopenthixol (LAI & oral)	Suicide death	QUIPS—low NOS—9
Taipale, 2021^78^	Finland, Sweden	BI, WI	Schizophrenia	Antipsychotics: Aripiprazole, clozapine, haloperidol, levomepromazine, olanzapine, perphenazine, quetiapine, risperidone (LAI & oral), zuclopenthixol (LAI)	Suicide attempt	QUIPS—low NOS—9
Tiihonen, 2012^79^	Finland	BI	Schizophrenia	Antidepressants: (unspecified)Benzodiazepines	Suicide death	QUIPS—low NOS—9
Toffol, 2015^80^	Finland	BI	Bipolar disorder	Antidepressants (unspecified), Antipsychotics (unspecified), BenzodiazepinesMood stabilisers: Lithium, valproic acid	Suicide death	QUIPS—low NOS—8
Tondo, 2020^81^	Multiple	BI	Bipolar disorder	Antidepressants (unspecified), antipsychotics (unspecified), lithium	Suicide attempt	QUIPS—low NOS—8
Tsai, 2016^82^	Taiwan	BI	Bipolar disorder	Antiepileptics: Carbamazepine, divalproex, lithium	Suicide attempt, suicide death	QUIPS—low NOS—9
Uwai, 2022^83^	Japan	BI	Bipolar disorder	Antipsychotics: Aripiprazole, carbamazepine, lithium, olanzapine, quetiapine, risperidone, valproate	Suicide attempt	QUIPS—low NOS—8
Valuck, 2016^84^	USA	BI	Depression	Antidepressants: SNRIs, SSRIs, TCAs	Suicide attempt	QUIPS—low NOS—9
Wimberley, 2017^85^	Denmark	BI	Schizophrenia	Antipsychotics: Clozapine	Self-harm, suicide death	QUIPS—low NOS—8
Xiang, 2008^86^	Hong Kong	BI	Schizophrenia	Benzodiazepines, clozapine	Suicide attempt	QUIPS—low NOS—8

All included studies sorted alphabetically by the first author.

BI, between-individual study design. WI, within-individual study design. QUIPS, Quality In Prognosis Studies tool. NOS, Newcastle-Ottawa Scale. SUD, substance use disorder. ADHD, attention deficit and hyperactivity disorder. FGA, first-generation antipsychotics. SGA, second-generation antipsychotics. SSRI, selective serotonin reuptake inhibitor. MAO, monoamine oxidase inhibitor. SNRI, serotonin-norepinephrine reuptake inhibitor. NaSSA, noradrenergic and specific serotonergic antidepressants. TCA, tricyclic antidepressant. LAI, long-acting injectable antipsychotic.

**Table 2:** Characteristics of pharmacological studies examining suicide outcomes by psychiatric disorder.

Diagnosis	Number of studies (k comparisons)	Mean follow-up time, weeks (years)	Mean participant age, years	Mean male %^a^	Number of medications	Number of countries
Schizophrenia spectrum disorders	19 (195)	384.7 (7.4)	40.8	54.5	44	11
Bipolar disorder	17 (107)	454.9 (8.7)	41.6	42.5	32	9
Depression	14 (53)	140.8 (2.7)	40.0	41.6	24	6
Personality disorders	5 (51)	357.7 (6.9)	29.7	19.0	37	3

a The proportion of male participants is estimated, as not all studies reported these data.

Seventy individual medications were examined, with the highest number in schizophrenia spectrum disorders and bipolar disorder (Table 2). In personality disorder, most studies were in people with borderline (emotionally unstable) personality disorder, where there was a lower mean age and proportion of men compared with other diagnostic groups. There were not enough studies in ADHD and substance use disorder to meet inclusion criteria, due to only one eligible study for each medication reported.

Results are presented for suicide attempts and suicide mortality separately. First, we present quantitative results for between-individual studies, and then findings from within-individual studies.

### Schizophrenia spectrum disorders

**Fig. 2:**
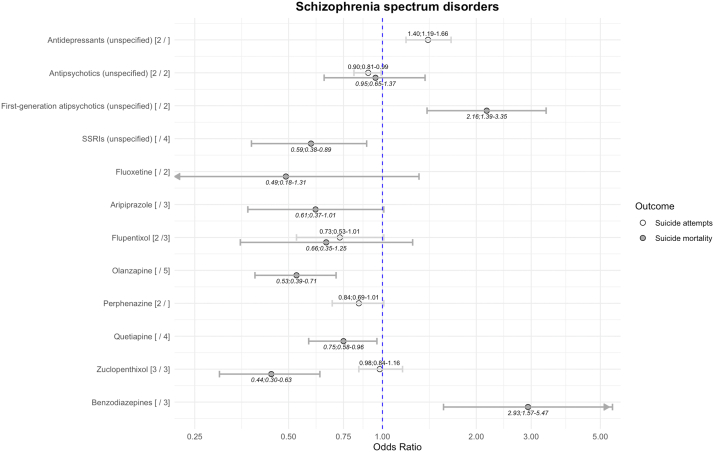
Risk of suicide-related outcomes in schizophrenia spectrum disorders using between-individual study designs. The numbers after the medication name reflect the number of comparisons in suicide attempts and suicide mortality. All results visualised have heterogeneity I^2^ < 50%. SSRIs, selective serotonin reuptake inhibitors.

Unspecified antipsychotics were associated with lower risk of suicide attempts (Fig. 2). In relation to individual medications, there was weak evidence for flupentixol and perphenazine being associated with lower risk of suicide attempts. Antidepressants were associated with higher risk of suicide attempts.

For suicide mortality, most second-generation antipsychotics were associated with lower risks. First-generation antipsychotics as a class were associated with elevated risks. Benzodiazepines were associated with higher risks of both outcomes. Risperidone was associated with lower risks of both suicide attempts (k = 3; OR = 0.61; 0.52–0.72; I^2^ = 57%) and suicide mortality (k = 4; OR = 0.55; 0.38–0.79; I^2^ = 64%) with high heterogeneity. Olanzapine was associated with lower risks of suicide attempts (k = 4; OR = 0.76; 0.60–0.98; I^2^ = 84%) with high heterogeneity, and clozapine was associated with lower risks of suicide mortality (k = 7; OR = 0.40; 0.36–0.60; I^2^ = 60%) with high heterogeneity. Overall, there was consistency in the direction of effects if the outcome was suicide attempts or suicide mortality.

We identified one within-individual study^78^ based on Nordic cohorts. Second generation antipsychotics were mostly associated with lower risk, whereas first-generation antipsychotics showed elevated risk of suicide-related outcomes. There was also one study each for antiepileptic/mood stabiliser gabapentin^49^ and ADHD medication methylphenidate,^72^ which did not show associations with suicide-related outcomes in any direction (p > 0.05).

### Bipolar disorder

**Fig. 3:**
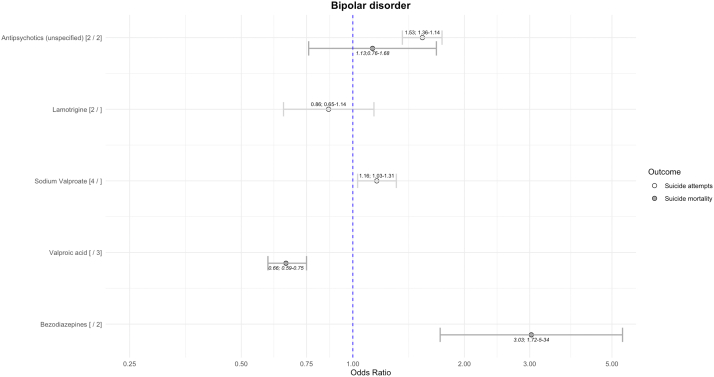
Risk of suicide-related outcomes in bipolar disorder using between-individual study design. The numbers after the medication name reflect the number of comparisons in suicide attempts and suicide mortality. All results visualised have heterogeneity I^2^ < 50%.

Unspecified antipsychotics and sodium valproate were associated with higher risk of suicide attempts (Fig. 3). Valproic acid was associated with lower risks of suicide mortality, and benzodiazepines were associated with elevated risks of suicide mortality. Unspecified mood stabilisers (k = 2; OR = 0.68; 0.56–0.83; I^2^ = 61%) and lithium (k = 6; OR = 0.38; 0.28–0.50; I^2^ = 67%) were associated with lower risks of suicide mortality.

For within-individual studies, lithium was associated with lower risk of suicide-related outcomes (k = 6; OR = 0.60; 0.44–0.82; I^2^ = 92%). Results for topiramate did not reach statistical significance (k = 2; OR = 0.60; 0.26–1.40; I^2^ = 0%). Findings for other medications showed no strong association with suicide-related outcomes and had high heterogeneity.

### Depression

**Fig. 4:**
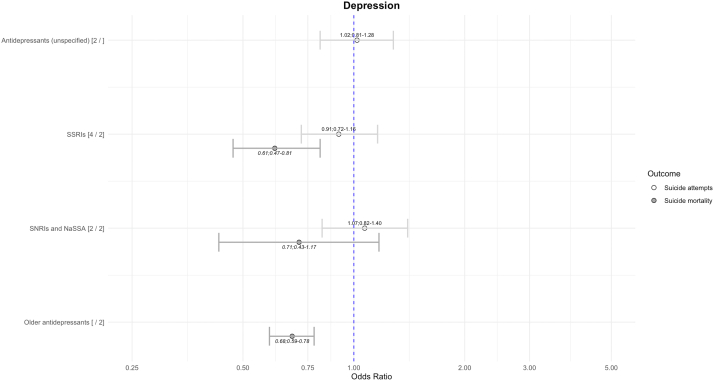
Risk of suicide-related outcomes in depression using between-individual study design. The numbers after the medication name reflect the number of comparisons in suicide attempts and suicide mortality. All results visualised have heterogeneity I^2^ < 50%. SSRIs, selective serotonin reuptake inhibitors. SNRI, Serotonin-norepinephrine reuptake inhibitor. NaSSA, noradrenergic and specific serotonergic antidepressants.

For suicide attempts, there was no strong evidence of association in any direction for antidepressants (Fig. 4). Antipsychotics as a class were associated with higher risk of suicide attempts (k = 3; OR = 2.43; 1.37–4.33; I^2^ = 72%), but with high heterogeneity.

For suicide mortality, SSRIs and older antidepressants (i.e. tricyclic antidepressants) were associated with reduced risks of suicide. Findings for other medications showed no strong association with suicide-related outcomes and had high heterogeneity.

There were three within-individual studies. One study^71^ found that methylphenidate was associated with lower risk of suicide-related outcomes in depression (OR = 0.45; 95% CI = 0.29–0.69). Other investigations showed lower risks of suicide for lithium^57^ and gabapentin.^49^

### Borderline and other personality disorders

Benzodiazepines were associated with elevated risk of suicide-related outcomes in personality disorders (with low/moderate heterogeneity). There was a link between benzodiazepines and suicide mortality (k = 2; OR = 4.29; 3.28–5.60; I^2^ = 0%).

In personality disorders, methylphenidate^72^ was associated with lower risk of suicide-related outcomes. Antipsychotics analysed in men and women separately^52^ were also associated with lower risk. One of the studies^51^ analysed suicide-related outcomes each month a year before and after quetiapine initiation. In comparison to 12 months before treatment, quetiapine was associated with higher risk of suicide-related outcomes, but there is evidence that suicide risk was growing for a year and then had a two-fold decrease in the first month after starting quetiapine. In another study,^59^ antidepressants and antipsychotics were mostly associated with elevated risk of suicide-related outcomes, except for antidepressant vortioxetine which was linked with lower risk. Methylphenidate and lisdexamfetamine were also associated with lower risk of suicide-related behaviour.

### Heterogeneity and sensitivity analysis

High between-study heterogeneity was observed for all suicide-related outcomes. One reason was outcome measurement. Suicidal behaviour included many outcomes that differed by intention and severity, including mortality and non-suicidal self-injury. Heterogeneity in the studies could be also explained by clinical factors: different medications with varying effects in one class, the difference in effect between diagnoses and the severity of the condition. For example, excluding a study that explored the effects of clozapine on suicide mortality in treatment-resistant schizophrenia lowered heterogeneity to less than 50% and resulted in a statistically significant finding.

We conducted a “leave-one-out” sensitivity analysis for the results reporting any suicide-related outcome with k ≥ 5. The results for all the explored medications were similar, but heterogeneity was lower. Since the meta-analysis was conducted in pre-specified subgroups with a small number of comparisons included, the results were derived with sufficient precision.

### Publication bias and evidence certainty

To assess possible publication bias, funnel plots were built for results with five or more studies, therefore analysing potential publication bias for studies on risks of suicide attempts (between-individual design) and suicide mortality for clozapine in people with schizophrenia spectrum disorder and for studies on risks of suicide attempts (within- and between-individual designs) and suicide mortality for lithium in people with bipolar disorder (Supplementary Materials S4). There was significant heterogeneity. Despite potential small-study effects, there was little evidence of publication bias for most funnel plots. However, there was evidence of some publication bias for suicide mortality for clozapine in schizophrenia spectrum disorder and for suicide attempts risk in within-individual studies for lithium in bipolar disorder, leading to more reporting of studies associated with lower risk of suicide-related outcomes for both funnel plots.

To assess the certainty of evidence, we used the GRADE framework (see Supplementary Materials S2). We have assessed the main findings of this review in terms of potential inconsistency, indirectness, and imprecision of evidence. For most medications, there was substantial heterogeneity, which indicates inconsistency. However, for the medications with lower heterogeneity (I^2^ < 50%) there was little concern for inconsistency. Due to the nature of observational studies, there was a small indirectness of interventions, since the main method for categorising a person as being under treatment was prescription data. The main concern in the certainty of the main findings was imprecision: many small studies with wide confidence intervals. Overall, quality of evidence is moderate.

## Discussion

This systematic review and meta-analysis examines the effect of psychotropic medications on suicide-related outcomes in observational studies. It summarises data on more than six million individuals, covering 48 studies and 70 individual medications across 5 main psychotropic classes. Medication effects by diagnostic category provided a framework with which to analyse the main findings.

In schizophrenia spectrum disorders, we found that second generation antipsychotics were linked to lower suicide risk, while lithium and valproic acid were associated with reduced risk in bipolar disorder. In depression, SSRIs and older tricyclic antidepressants were associated with protective effects for suicide mortality in community-based adults. Benzodiazepines, however, were linked to increased suicide risk in schizophrenia spectrum disorders, bipolar disorder and personality disorders.

These results have potential clinical implications for management of people with high risks of suicide-related outcomes. The findings, taken together, suggest that suicide prevention strategies should consider the appropriate use of prescribed medications in people with psychiatric disorders. In addition, individuals prescribed benzodiazepines require follow-up and periodic medication reviews to avoid chronicity and potential harms. At the same time, the review findings are not causal, and need triangulation using other research designs, including target trial emulation and mechanistic preclinical studies. Trials with suicide outcomes may not be feasible, and proxy outcomes such as suicide attempts can be considered with caution. The review also highlights the importance of diagnosis in people with mental disorders—the effects of medication clearly differed by main diagnostic category.

Some of these findings triangulate with trial data. Lithium has been associated with lower risk of suicide,^6^^,^^12^ and benzodiazepines linked to elevated risk in randomised controlled trials.^87^ The evidence on SSRIs has been inconsistent: while a recent meta-analysis of RCTs suggests reduced suicidal behaviour,^88^ older trial data provide evidence of either elevated risk of suicide attempts^89^ or no effect on any suicide-related outcomes,^90^ and our review supports the former. One previous review found that different SSRIs have varying effects on suicide ideation,^6^ but not on other suicide-related outcomes. For antipsychotics, clozapine has consistently been associated with lower risk of suicide attempts: in a systematic review of 51 studies, including 9 clinical trials,^91^ clozapine showed an 86% reduction of suicide attempts in comparison to placebo, and compared with olanzapine. Our results suggest that other second-generation antipsychotics are associated with reduced risk, particularly for suicide deaths in schizophrenia. In addition, in bipolar disorder, we found valproic acid was associated with reduced risk of suicide mortality, although current guidelines advise against its use due to concerns about long-term adverse effects.^92^

Suicide attempts and deaths are relatively rare outcomes, including among individuals with diagnosed psychiatric disorders, and they typically are not the primary target of most treatments. Although certain medications show protective associations with suicide-related outcomes, they are unlikely to prevent all such events. Pharmacological interventions should therefore be implemented alongside psychosocial treatments and other risk management strategies for suicide prevention. Furthermore, the development and validation of prognostic risk models are necessary to provide more individualised, consistent and transparent estimates of risk,^93^^,^^94^ enabling more effective targeting of preventive resources and complementing decision-making in clinical practice.

We found differences in associations with suicide risk when comparing within-individual and between-individual study designs. Within-individual studies often reported increased suicide risk during treatment, which likely reflects the fact that treatment often begins when symptoms worsen. Many studies used long follow-up periods (six months or more), during which risk levels can change significantly. Some studies^51^^,^^95^ show that suicide risk tends to rise in the year before treatment starts and peaks just before treatment begins. After treatment starts, suicide risk usually drops but may remain higher than a baseline some months before treatment started.

Between-individual studies are affected by confounding by indication. For example, medications such as first-generation antipsychotics are typically prescribed to people with more severe illness or those who have not responded or partially responded to other treatments. These patients are already at higher risk of suicide, which may explain why some medications appear to be linked to poorer outcomes in these studies.^96^ In addition to confounding, these studies are vulnerable to other biases—such as immortal time bias, and prevalent user bias, which are due to a misalignment in treatment initiation and start of follow-up.^97^ These biases can result in misleading associations and further limit the validity of findings. The target trial framework can improve the quality of between-individual analyses and help prevent some common biases. However, the possibility of residual confounding remains.^98^

This review included large, high-quality studies. By looking at both within-individual and between-individual designs, we were able to identify sources of heterogeneity. Limitations should be considered. Most medications were only studied in a few studies, which limited analysis of publication bias or between-study heterogeneity. Many investigations were excluded because they did not report underlying diagnoses, and outcome definitions varied: some focused on suicide attempts or non-suicidal self-injury, while others included deaths. Suicide-related adverse effects may not have been captured due to healthcare registries rarely reporting them. Inclusion of such events would likely skew the data towards higher odds of suicide-related outcomes, especially non-fatal. Although there was a very high level of agreement between reviewers regarding screening decisions, only 20% of studies were screened independently. Since we excluded studies conducted in selected populations (e.g. people in prison, specific age groups), this could potentially limit our findings, such as in children and adolescents. Future reviews should consider comparing suicide-related outcomes in different age and socio-demographic groups. As suicides were very rare, and the differences between outcomes were minimal, this did not clearly influence findings. All studies were conducted in cohorts from high- and upper-middle-income countries.

Future studies should consider more targeted designs, especially within-individual comparisons and target trial emulations, with clearly defined outcomes. Pharmacoepidemiological studies for suicide prevention should avoid combining mental health diagnoses. More research is needed in substance use disorders, which were not included in the current review despite being at high risk, in low- and middle-income countries, and on medications where uncertainty remains, such as second-generation antipsychotics in bipolar disorder, or SNRIs in depression. There was some consistency in the direction of effects whether the outcome was suicide attempts/self-harm or suicide mortality, suggesting that combining these outcomes can be considered when considering triangulation of these findings with other study designs.

In summary, we found that some psychotropic medications were associated with reduced risk of suicide-related outcomes, although the magnitude and direction of these effects differed across psychiatric diagnoses, including schizophrenia spectrum disorders, bipolar disorder, depression, and personality disorder.

## Contributors

SK completed data screening, data extraction, data curation, statistical analysis, data visualisation, and writing (original draft and editing). CE reviewed the abstract and title screening, reviewed data extraction, and assisted in writing (review). GS, DY, and ZC aided in conceptualisation, supervision, and writing (review and editing). SF led on conceptualisation, supervision, and writing (review and editing). SK and CE directly accessed and verified the underlying data reported in the manuscript. All authors had full access to all the data in the study and accept responsibility to submit for publication.

## Data sharing statement

Individual participant data are not available. The study protocol was published with PROSPERO (CRD42024515794) and is available at https://www.crd.york.ac.uk/PROSPERO/view/CRD42024515794.

## Declaration of interests

ZC has received grants from Swedish Research Council and lecture honoraria from Take Pharmaceuticals. SK, CE, GS, DY, and SF declare no competing interests.

## Appendix A Supplementary data

Supplemenrary Materials
