# Corrigendum to “Effect of psychotropic medications on suicide-related outcomes: a systematic review and meta-analysis of observational studies”

**DOI:** 10.1016/j.eclinm.2026.103901

**Published:** 2026-04-09

**Authors:** Stefaniya Kozhevnikova, Christina Emilian, Giulio Scola, Zheng Chang, Denis Yukhnenko, Seena Fazel

**Affiliations:** a Department of Psychiatry, University of Oxford, Oxford, United Kingdom; b Department of Social Policy and Intervention, University of Oxford, Oxford, United Kingdom; c Department of Medical Epidemiology and Biostatistics, Karolinska Institutet, Stockholm, Sweden

The following should be added to Declaration of interests:

SF has received an honorarium from Angelini Pharma for speaking at a conference on epilepsy care and no research funding relevant to this review.

This declaration was notified at the proof stage.

