# Supplementary material for: Effect of psychotropic medications on suicide-related outcomes: a systematic review and meta-analysis of observational studies
Source: eClinicalMedicine. 2026 Feb 21;93:103800. doi: 10.1016/j.eclinm.2026.103800 (PMC12945523; doi:10.1016/j.eclinm.2026.103800)
Supplement: Supplemenrary Materials [file mmc1.pdf]

Supplementary Materials

S1. Search strategy

(((self-harm or self harm or selfharm or suicid*) and (antidepressant* or SSRI* or TCA* or SNRI* or NASSA* or SARI* or MAO* or anti-anxiety or tranquil* or benzodiazepin* or anxiolytic* or anxiolitic* or antipsychotic* or neuroleptic* or mood stabili* or antiepileptic* or acamprosate or agomelatine or alprazolam or amisulpride or amitriptyline or amoxapine or amphetamine or aripiprazole or armodafinil or asenapine or atomoxetine or benperidol or buprenorphine or bupropion or buspirone or cariprazine or chlordiazepoxide or chlorpromazine or citalopram or clomipramine or clonazepam or clonidine or clorazepate or clozapine or cyamemazine or desipramine or desvenlafaxine or dexamfetamine or diazepam or disulfiram or dosulepin or dothiepin or doxepin or duloxetine or escitalopram or fluoxetine or flupentixol or fluvoxamine or guanfacine or haloperidol or hydroxyzine or imipramine or isocarboxazid or lamotrigine or levomepromazine or lisdexamfetamine or lithium or lofepramine or lofexidine or loflazepate or lorazepam or loxapine or lurasidone or maprotiline or melatonin or methadone or methylphenidate or mianserin or mirtazapine or moclobemide or modafinil or nalmefene or naltrexone or nefazodone or nortriptyline or olanzapine or oxazepam or paliperidone or paroxetine or phenelzine or pimozide or prazosin or prochlorperazine or propranolol or quetiapine or reboxetine or risperidone or selegiline or sertraline or sulpiride or tianeptine or tranylcypromine or trazodone or trifluoperazine or trimipramine or valproate or varenicline or venlafaxine or vilazodone or vortioxetine or zuclopenthixol)).ab. and (observational or target trial or cohort or case-control or case control).af.) not (systematic review or meta-analysis or meta-review or umbrella review).ti. not (psilocybin* or ketamine or esketamine or psychodelic* or psylocibin*).ab

S2. Results by diagnosis

Table S2.1 Effects of medications when used in individuals with schizophrenia spectrum disorders. Bold text – the result is statistically significant.

Outcomes	Suicide attempt					Suicide mortality				
Study design	Between-individual studies									
	k studies	OR	CI 95%	I^2	Certainty of evidence	k studies	OR	CI 95%	I^2	Certainty of evidence
Antidepressants (unspecified)	2	**1.40**	**1.19-1.66**	**0%**	High	3	0.77	0.25-2.37	90%	Moderate (inconsistency)
SSRIs						4	**0.59**	**0.38-0.89**	12%	High
Citalopram						2	0.50	0.15-1.65	77%	Moderate (inconsistency)
Fluoxetine						2	0.49	0.18-1.31	0%	Moderate (imprecision)
Antipsychotics (unspecified)	2	**0.90**	**0.81-0.99**	**0%**	High	2	0.95	0.65-1.37	0%	High
First-generation antipsychotics (unspecified)						**2**	**2.16**	**1.39-3.35**	0%	High
Second-generation antipsychotics (unspecified)						3	0.65	0.27-1.53	73%	Moderate (inconsistency)
Aripiprazole	2	0.81	0.45-1.48	87%	Moderate (inconsistency)	3	0.61	0.37-1.01	0%	Moderate (imprecision)
Chlorprothixene						2	1.44	0.42-4.97	90%	Moderate (inconsistency)
Clozapine	6	0.62	0.37-1.02	94%	Moderate (inconsistency)	**7**	**0.40**	**0.36-0.60**	**60%**	Moderate (inconsistency)
Flupentixol	2	0.73	0.53-1.01	0%	High	3	0.66	0.35-1.25	28%	Moderate (imprecision)
Haloperidol	4	1.16	0.73-1.83	89%	Moderate (inconsistency)	4	0.82	0.49-1.38	65%	Moderate (inconsistency)
Olanzapine	4	**0.76**	**0.60-0.98**	**84%**	Moderate (inconsistency)	5	**0.53**	**0.39-0.71**	34%	High
Perphenazine	2	0.84	0.69-1.01	20%	High	4	0.72	0.45-1.16	65%	Moderate (inconsistency)
Risperidone	3	**0.61**	**0.52-0.72**	**57%**	Moderate (inconsistency)	**4**	**0.55**	**0.38-0.79**	**64%**	Moderate (inconsistency)
Levomepromazine						2	1.14	0.54-2.43	71%	Moderate (inconsistency)

Quetiapine	3	1.12	0.74-1.70	93%	Moderate (inconsistency)	4	**0.75**	**0.58-0.96**	0%	High
Zuclopenthixol	3	0.98	0.84-1.16	0%	High	3	**0.44**	**0.30-0.63**	0%	High
Benzodiazepines	3	2.78	0.91-8.47	93%	Moderate (inconsistency)	3	**2.93**	**1.57-5.47**	14%	High
Lithium						2	0.78	0.36-1.66	100%	Moderate (inconsistency)

Table S2.2. Effects of individual medications when used in individuals with bipolar disorder. Bold text – the result is statistically significant.

Outcomes	Suicide attempt										Suicide mortality				
Study design	Within-individual studies					Between-individual studies									
	k studies	OR	CI 95%	I^2	Certainty of evidence	k studies	OR	CI 95%	I^2	Certainty of evidence	k studies	OR	CI 95%	I^2	Certainty of evidence
Mood stabilisers (unspecified)											**2**	**0.68**	**0.56-0.83**	**61%**	Moderate (inconsistency)
Lithium	**6**	**0.60**	**0.44-0.82**	**92%**	Moderate (inconsistency)	5	0.86	0.67-1.11	89%	Moderate (inconsistency)	**6**	**0.38**	**0.28-0.50**	**67%**	Moderate (inconsistency)
Antipsychotics (unspecified)	3	1.38	0.69-2.73	97%	Moderate (inconsistency)	2	**1.53**	**1.36-1.74**	**0%**	High	2	1.13	0.76-1.68	48%	Moderate (imprecision)
Antidepressants (unspecified)	2	0.71	0.42-1.20	85%	Moderate (inconsistency)										
Antiepileptics (unspecified)	2	0.39	0.10-1.59	97%	Moderate (inconsistency)	2	1.76	0.40-7.71	90%	Moderate (inconsistency)					
Carbamazepine	2	0.42	0.14-1.29		Moderate (imprecision)	3	1.23	0.59-2.56	72%	Moderate (inconsistency)					
Divalproex															
Gabapentin	2	0.98	0.36-2.66												
Lamotrigine	2	0.56	0.21-1.52	89%	Moderate (inconsistency)	2	0.86	0.65-1.14	0%	Moderate (imprecision)					
Topiramate	2	0.60	0.26-1.40	0%	Moderate (imprecision)										
Sodium Valproate	2	0.87	0.60-1.25	81%	Moderate (inconsistency)	4	**1.16**	**1.03-1.31**	13%	High					
Valproic Acid											3	**0.66**	**0.59-0.75**	**0%**	**High**
Benzodiazepines											2	**3.03**	**1.72-5.34**	**0%**	**High**

Table S2.3. Effects of medications when used in individuals with depression. Bold text – the result is statistically significant.

Outcomes	Suicide attempt					Suicide mortality				
Study design	Between-individual studies									
	k studies	OR	CI 95%	I^2	Certainty of evidence	k studies	OR	CI 95%	I^2	Certainty of evidence
Antidepressants unspecified	2	1.02	0.81-1.28	0%	Moderate (imprecision)	3	0.69	0.33-1.47	92%	Moderate (imprecision)
SSRIs	4	0.91	0.72-1.16	0%	High	2	**0.61**	**0.47-0.81**	**23%**	**High**
New non-SSRIs	2	1.07	0.82-1.40	0%	Moderate (imprecision)	2	0.71	0.43-1.17	43%	Moderate (imprecision)
Older antidepressants	4	0.60	0.21-1.70	64%	Moderate (imprecision)	2	**0.68**	**0.59-0.78**	**0%**	**High**
Antipsychotics overall	3	**2.43**	**1.37-4.33**	**72%**	Moderate (inconsistency)					
Benzodiazepines						2	2.00	0.94-4.22	71%	Moderate (imprecision)

Table S2.4 Effects of individual medications when used in individuals with personality disorders. Bold text – the result is statistically significant.

Outcomes	Suicide attempt					Suicide mortality				
Study design	Within-individual studies									
	k studies	OR	CI 95%	I^2	Certainty of evidence	k studies	OR	CI 95%	I^2	Certainty of evidence
Methylphenidate	2	0.59	0.28 -1.28	86%	Low (inconsistency, imprecision)					
Antipsychotics overall	3	0.97	0.67 -1.40	99%	Moderate (inconsistency)					
Quetiapine	2	1.05	0.92 -1.19	14%	High					
Benzodiasepines						2	**4.29**	**3.28-5.60**	0%	High

S3. Studies excluded at the full-text screening stage

Table S3. All excluded studies with the exclusion reason, sorted by the first author in an alphabetical order. Some of the papers would be excluded with multiple reasons, they appear in either a more appropriate category or the one that was noticed first.

First author	Year	Title	DOI or link
**Diagnosis**			
Andersohn	2010	Use of antiepileptic drugs in epilepsy and the risk of self-harm or suicidal behavior	https://dx.doi.org/10.1212/WNL.0b013e3181ea157e
Bharat	2024	Association of Opioid Analgesics, Benzodiazepines, Gabapentinoids, and Opioid Agonist Treatment With Mortality Among Individuals With Opioid Dependence	https://doi.org/10.1016/j.drugalcdep.2023.110845
Boggs	2020	Association between suicide death and concordance with benzodiazepine treatment guidelines for anxiety and sleep disorders	https://www.sciencedirect.com/science/article/abs/pii/S0163834319303214?via%3Dihub
Chang	2020	Medication for Attention-Deficit/Hyperactivity Disorder and Risk for Suicide Attempts	10.1016/j.biopsych.2019.12.003
Grimaldi-Bensouda	2017	Antiepileptic drugs and risk of suicide attempts: a case–control study exploring the impact of underlying medical conditions	https://dx.doi.org/10.1002/pds.4160
Irigoyen	2019	Predictors of re-attempt in a cohort of suicide attempters: A survival analysis	10.1016/j.jad.2018.12.050
Jakobsen	2023	Risk of repeated suicide attempt after redeeming prescriptions for antidepressants: a register-based study in Denmark	https://dx.doi.org/10.1017/S0033291722002719

Katz	2020	Psychotropic medication use before and after suicidal presentations to the emergency department: A longitudinal analysis.	https://dx.doi.org/10.1016/j.genhosppsych.2018.10.003
Lagerberg	2022	Selective serotonin reuptake inhibitors and suicidal behaviour: a population-based cohort study	https://dx.doi.org/10.1038/s41386-021-01179-z
Leon	2010	Antidepressants and risks of suicide and suicide attempts a 27-year observational study.	https://dx.doi.org/10.4088/JCP.10m06552
Leon	2012	Two propensity score-based strategies for a three-decade observational study: Investigating psychotropic medications and suicide risk	https://dx.doi.org/10.1002/sim.5339
Molero	2018	Medications for alcohol and opioid use disorders and risk of suicidal behavior, accidental overdoses, and crime.	https://dx.doi.org/10.1176/appi.ajp.2018.17101112
Molero	2019	Associations between gabapentinoids and suicidal behaviour, unintentional overdoses, injuries, road traffic incidents, and violent crime: Population based cohort study in Sweden	https://dx.doi.org/10.1136/bmj.l2147
Neutel	1997	Risk of suicide attempts after benzodiazepine and/or antidepressant use	https://dx.doi.org/10.1016/S1047-2797%2897%2900126-9
Ohlund	2020	Suicidal and non-suicidal self-injurious behaviour in patients with bipolar disorder and comorbid attention deficit hyperactivity disorder after initiation of central stimulant treatment: a mirror-image study based on the LiSIE retrospective cohort	https://dx.doi.org/10.1177/2045125320947502
Olesen	2010	Antiepileptic drugs and risk of suicide: a nationwide study.	10.1002/pds.1932
Padmanathan	2022	Self-harm and suicide during and after opioid agonist treatment among primary care patients in England: a cohort study	10.1016/S2215-0366(21)00392-8

Park	2015	Identifying clinical correlates for suicide among epilepsy patients in South Korea: A case-control study	https://dx.doi.org/10.1111/epi.13226
Rahman	2014	Risk factors for suicidal behaviour in individuals on disability pension due to common mental disorders - A nationwide register-based prospective cohort study in Sweden	https://dx.doi.org/10.1371/journal.pone.0098497
Schuerch	2016	Impact of varying outcomes and definitions of suicidality on the associations of antiepileptic drugs and suicidality: Comparisons from UK Clinical Practice Research Datalink (CPRD) and Danish national registries (DNR)	http://onlinelibrary.wiley.com/journal/10.1002/(ISSN)1099-1557
Siffel	2020	Cohort study evaluating suicidal behavior associated with ADHD stimulant therapies	https://www.sciencedirect.com/science/article/pii/S016503271831509X#tbl0003
Stricker	2022	General practice database on mortality in adults on methylphenidate: cohort study	https://dx.doi.org/10.1136/bmjopen-2021-057303
Takeuchi	2017	The relationship between psychotropic drug use and suicidal behavior in Japan: Japanese adverse drug event report.	https://dx.doi.org/10.1055/s-0042-113468
Tournier	2023	Risk of suicide attempt and suicide associated with benzodiazepine: A nationwide case crossover study.	https://dx.doi.org/10.1111/acps.13582
Xi	2022	Impact of social disparities on risk factors for suicidal ideation and suicide attempt among commercially insured youth and adults in the US.	https://www.ncbi.nlm.nih.gov/pmc/articles/PMC10011396/
**Study design**			
Amendola	2021	Did the introduction and increased prescribing of antidepressants lead to changes in long-term trends of suicide rates?	https://dx.doi.org/10.1093/eurpub/ckaa204

Angst	2005	Suicide in 406 Mood-Disorder Patients With and Without Long-Term Medication: A 40 to 44 Years' Follow-Up.	https://dx.doi.org/10.1080/13811110590929488
Bagley	2017	Association between psychostimulant agents and suicide related events in individuals with Attention-Deficit Hyperactivity Disorder.	
Baldessarini	2003	Lithium treatment and suicide risk in major affective disorders: Update and new findings	
Bell	2008	Health care utilization and morbidity associated with methadone and buprenorphine treatment	http://www.atforum.com/pdf/europad/HeroinAdd10-2.pdf
Bocchetta	1998	Suicidal Behavior On and Off Lithium Prophylaxis in a Group of Patients With Prior Suicide Attempts	
Born	2005	Newer prophylactic agents for bipolar disorder and their influence on suicidality	https://dx.doi.org/10.1080/13811110590929541
Brodersen	2000	Sixteen-year mortality in patients with affective disorder commenced on lithium	https://dx.doi.org/10.1192/bjp.176.5.429
Caplehorn	1996	Methadone maintenance and addicts' risk of fatal heroin overdose	http://dx.doi.org/10.3109/10826089609045806
Carvalho	2024	Mortality and lithium-protective effects after first-episode mania diagnosis in bipolar disorder: A nationwide retrospective cohort study in Taiwan.	https://dx.doi.org/10.1159/000535777
Castelpietra	2016	Diagnoses and prescriptions of antidepressants in suicides: Register findings from the Friuli Venezia Giulia Region, Italy, 2002-2008	https://dx.doi.org/10.3109/13651501.2016.1149196

Castelpietra	2008	Antidepressant use and suicide prevention: a prescription database study in the region Friuli Venezia Giulia, Italy	
Chan	2024	Mortality risk and mood stabilizers in bipolar disorder: a propensity-score-weighted population-based cohort study in 2002-2018.	https://dx.doi.org/10.1017/S2045796024000337
Chawla	2022	Assessment of lethality and its clinical correlates in suicide attempters with mood disorders	10.4103/ipj.ipj_251_21
Cheung	2015	Antidepressant use and the risk of suicide: A population-based cohort study	https://dx.doi.org/10.1016/j.jad.2014.12.032
Collazo	2022	A Retrospective Cohort Study on the Increasing Trend of Suicide Ideations and Risks in an Opioid-Dependent Population of Puerto Rico 2015-2018	https://dx.doi.org/10.1007/s10903-021-01310-8
Collins	2008	Divalproex, lithium and suicide among Medicaid patients with bipolar disorder.	https://dx.doi.org/10.1016/j.jad.2007.07.014
Conner	2007	Suicide attempts among individuals with opiate dependence: The critical role of belonging	https://dx.doi.org/10.1016/j.addbeh.2006.09.012
Connery	2018	Microscopes and telescopes: The societal impact of substance use disorder treatment.	https://dx.doi.org/10.1176/appi.ajp.2018.18060694
Coryell	2001	Lithium and suicidal behavior in major affective disorder: A case-control study	https://dx.doi.org/10.1034/j.1600-0447.2001.00338.x
Cougnard	2009	Impact of antidepressants on the risk of suicide in patients with depression in real-life conditions: a decision analysis model	

Coull	2000	Post-mortem studies of brain phosphatidylinositol hydrolysis in depression and the effect of antidepressant treatment	10.1017/S1461145700001851
Coupland	2015	Antidepressant use and risk of suicide and attempted suicide or self harm in people aged 20 to 64: cohort study using a primary care database	
Courtet	2010	Suicidality: risk factors and the effects of antidepressants. The example of parallel reduction of suicidality and other depressive symptoms during treatment with the SNRI, milnacipran	https://dx.doi.org/10.2147/NDT.S11774
Courtet	2017	Antidepressants and suicide risk in depression.	https://dx.doi.org/10.1002/wps.20460
Crocq	2010	Suicide attempts in a prospective cohort of patients with schizophrenia treated with sertindole or risperidone	https://dx.doi.org/10.1016/j.euroneuro.2010.09.001
De Hert	2010	Do antipsychotic medications reduce or increase mortality in schizophrenia? A critical appraisal of the FIN-11 study.	https://dx.doi.org/10.1016/j.schres.2009.12.029
De la Fuente-Sandoval	2019	P.614 Depression and suicidal ideation in treatment-resistent depression patients in latam: cross-sectional analysis from the multicentre, prospective, observational TRAL study	https://dx.doi.org/10.1016/j.euroneuro.2019.09.598
Delapaz	2021	An emulation of randomized trials of administrating antipsychotics in ptsd patients for outcomes of suicide-related events	https://dx.doi.org/10.3390/jpm11030178
Denee	2024	Impact of moderate-to-high-suicide-intent in major depressive disorder: a retrospective cohort study on patient characteristics and healthcare resource utilisation in England.	https://dx.doi.org/10.1186/s12888-024-05961-3
Fedyszyn	2014	Suicidal behaviours during treatment for first-episode psychosis: Towards a comprehensive approach to service-based prevention	https://dx.doi.org/10.1111/eip.12084

Fernandez-Miranda	2016	Clinical and functional outcomes of patients with severe schizophrenia undergoing comprehensive treatment: A 6-year follow-up	https://dx.doi.org/10.1016/j.eurpsy.2016.01.629
Fernandez-Miranda	2020	High Doses of Second-Generation Long-Acting Antipsychotics in the Treatment of Patients with Severe Resistant Schizophrenia: A Six-Year Mirror-Image Study	https://dx.doi.org/10.5455/PCP.20201011042823
Fernandez-Miranda	2022	The Use of Second-Generation Antipsychotics in Patients with Severe Schizophrenia in the Real World: The Role of the Route of Administration and Dosage-A 5-Year Follow-Up.	https://dx.doi.org/10.3390/biomedicines11010042
Fleischhacker	2014	Completed and attempted suicides among 18,154 subjects with schizophrenia included in a large simple trial	https://dx.doi.org/10.4088/jcp.13m08563
Fond	2023	How can we improve the care of patients with schizophrenia in the real-world? A population-based cohort study of 456,003 patients	https://dx.doi.org/10.1038/s41380-023-02154-4
Garcia-Carmona	2020	Long-Acting Injectable Antipsychotics: Analysis of Prescription Patterns and Patient Characteristics in Mental Health from a Spanish Real-World Study	https://dx.doi.org/10.1007/s40261-020-00913-7
Garcia-Carmona	2021	Evaluation of long-acting injectable antipsychotics with the corresponding oral formulation in a cohort of patients with schizophrenia: a real-world study in Spain	10.1097/YIC.0000000000000339
Goldstein	1995	Heroin addicts and methadone treatment in Albuquerque: A 22-year follow-up	https://dx.doi.org/10.1016/0376-8716%2895%2901205-2
Gonzalez-Pinto	2006	Suicidal risk in bipolar I disorder patients and adherence to long-term lithium treatment	

Goodwin	2003	Suicide Risk in Bipolar Disorder during Treatment with Lithium and Divalproex	https://dx.doi.org/10.1001/jama.290.11.1467
Haukka	2009	Antidepressant use and mortality in Finland: A register-linkage study from a nationwide cohort	https://dx.doi.org/10.1007/s00228-009-0616-9
Hayes	2016	Self-harm, Unintentional Injury, and suicide in bipolar disorder during maintenance mood stabilizer treatment a UK population-based electronic health records study	ttps://dx.doi.org/10.1001/jamapsychiatry.2016.0432
Henriksson	2011	Suicides are seldom prescribed antidepressants: findings from a prospective prescription database in Jamtland county, Sweden, 1985-95	
Hesdorffer	2016	Occurrence and recurrence of attempted suicide among people with epilepsy	https://dx.doi.org/10.1001/jamapsychiatry.2015.2516
Huang	2016	Comparison of Long-Acting Injectable Antipsychotics with Oral Antipsychotics and Suicide and All-Cause Mortality in Patients with Newly Diagnosed Schizophrenia	https://dx.doi.org/10.1001/jamanetworkopen.2021.8810
Isaacson	2009	Decrease in suicide among the individuals treated with antidepressants: A controlled study of antidepressants in suicide, Sweden 1995-2005.	https://dx.doi.org/10.1111/j.1600-0447.2009.01344.x
Jalbert	2016	Channeling bias in a comparative effectiveness study of a newly launched antipsychotic	https://dx.doi.org/10.1002/pds.4070
Jiang	2021	Suicide prediction among men and women with depression: A population-based study	https://dx.doi.org/10.1016/j.jpsychires.2021.08.003
Jick	2004	Antidepressants and the Risk of Suicidal Behaviors	

Joffe	2004	Does lithium save lives?	
Joseph	2022	Association between mirtazapine use and serious self-harm in people with depression: an active comparator cohort study using UK electronic health records	https://dx.doi.org/10.1136/ebmental-2021-300355
Kamat	2014	Association between antidepressant prescribing and suicide rates in OECD countries: An ecological study.	https://dx.doi.org/10.1055/s-0033-1357183
Kapur	1992	Antidepressant medications and the relative risk of suicide attempt and suicide	https://dx.doi.org/10.1001/jama.268.24.3441
Kelty	2018	Morbidity and mortality in opioid dependent patients after entering an opioid pharmacotherapy compared with a cohort of non-dependent controls	https://dx.doi.org/10.1093/pubmed/fdx063
Kim	2024	Risk factors of reattempt among suicide attempters in South Korea: A nationwide retrospective cohort study.	https://dx.doi.org/10.1371/journal.pone.0300054
Leith	2019	The association between gabapentin and suicidality in bipolar patients	https://dx.doi.org/10.1097/YIC.0000000000000242
Leppein	2023	Newer Antiseizure Medications and Suicidality: Analysis of the Food and Drug Administration Adverse Event Reporting System (FAERS) Database	https://dx.doi.org/10.1007/s40261-023-01272-9
Machado	2011	Suicidal risk and suicide attempts in people treated with antiepileptic drugs for epilepsy	https://dx.doi.org/10.1016/j.seizure.2010.12.010
Martinez	2005	Antidepressant treatment and the risk of fatal and non-fatal self harm in first episode depression: Nested case-control study.	https://dx.doi.org/10.1136/bmj.330.7488.389

Maust	2023	Benzodiazepine Discontinuation and Mortality Among Patients Receiving Long-Term Benzodiazepine Therapy.	10.1001/jamanetworkopen.2023.48557
McGirr	2006	Risk factors for completed suicide in schizophrenia and other chronic psychotic disorders: A case-control study	10.1016/j.schres.2006.02.025
Miller	2014	Antidepressant class, age, and the risk of deliberate self-harm: A propensity score matched cohort study of SSRI and SNRI users in the USA.	https://dx.doi.org/10.1007/s40263-013-0120-8
Miller	2014	Antidepressant Dose, Age, and the Risk of Deliberate Self-harm	10.1001/jamainternmed.2014.1053
Montastruc	2019	Association of Aripiprazole with the risk of psychiatric hospitalization, self-harm, or suicide	https://jamanetwork.com/journals/jamapsychiatry/fullarticle/2722562
Moon	2020	Premature mortality and causes of death of people with epilepsy in South Korea	
Moore	2023	Association between psychotropic drug prescription and suicide rates in Scotland: population study	https://dx.doi.org/10.1192/bjb.2021.88
Mulleroerlinghausen	1992	SUICIDES AND PARASUICIDES IN A HIGH-RISK PATIENT GROUP ON AND OFF LITHIUM LONG-TERM MEDICATION	10.1016/0165-0327(92)90084-J
Nakagawa	2007	Association of suicide and antidepressant prescription rates in Japan, 1999-2003.	https://dx.doi.org/10.4088/JCP.v68n0613
Nettelbladt	2007	Suicide rates in the Lundby cohort before and after the introduction of tricyclic antidepressant drugs	
Newhouse	2021	Using an Observational Dataset to Study the Association of Antidepressant Treatment Use with Suicidal Ideation, Self-Harm	

		and Death by Suicide : a Retrospective Study of Patients, Who Received a Clinical Diagnosis of Depression, in Secondary Psychiatric Care	
Nielsen	2018	Second-generation LAI are associated to favorable outcome in a cohort of incident patients diagnosed with schizophrenia	https://dx.doi.org/10.1016/j.schres.2018.07.020
Lahteenvuo	2022	Association of opioid agonist treatment with all-cause mortality and specific causes of death among people with opioid dependence: A systematic review and meta-analysis	
Novick	2010	Predictors and clinical consequences of non-adherence with antipsychotic medication in the outpatient treatment of schizophrenia	https://dx.doi.org/10.1016/j.psychres.2009.05.00
Osler	2019	P.219 Antidepressant medication, suicidal behaviour and violent crime in a cohort of Danish patients with affective disorders	https://dx.doi.org/10.1016/j.euroneuro.2019.09.262
Ouazana-Vedrines	2022	Outcomes associated with antidepressant treatment according to the number of prescriptions and treatment changes: 5-year follow-up of a nation-wide cohort study	https://dx.doi.org/10.3389/fpsyt.2022.923916
Park	2016	Suicidal thoughts/acts and clinical correlates in patients with depressive disorders in Asians: Results from the REAP-AD study.	https://dx.doi.org/10.1017/neu.2016.27
Perucci	1991	MORTALITY OF INTRAVENOUS-DRUG-USERS IN ROME - A COHORT STUDY	10.2105/AJPH.81.10.1307
Reselan	2006	Relationship between antidepressant sales and secular trends in suicide rates in the Nordic countries	

Ridout		Computational Strategies to Tailor Existing Interventions for First Major Depressive Episodes to Inform and Test Personalized Interventions	
Rihman	2015	Treatment of bipolar disorder with lamotrigine -- relapse rate and suicidal behaviour during 6 month follow-up	
Rissanen	2014	ANTIPSYCHOTICS AND ANTIDEPRESSANTS AND THEIR ASSOCIATIONS WITH SUICIDAL IDEATION - THE NORTHERN FINLAND BIRTH COHORT 1966	10.1016/S0920-9964(14)71013-8
Rohde	2024	A target trial emulation comparing the antidepressant effectiveness of Selective Serotonin Reuptake Inhibitors (SSRIs) highlighting the importance of patent-related confounding by indication.	https://dx.doi.org/10.1111/acps.13729
Rubino	2007	Risk of suicide during treatment with venlafaxine, citalopram, fluoxetine, and dothiepin: Retrospective cohort study	https://dx.doi.org/10.1136/bmj.39041.445104.BE
Sacchi	2021	Impact of antidepressant prescriptions on suicidal behavior in times of severe financial strain	https://dx.doi.org/10.1097/NMD.0000000000001336
Sani	2011	Suicide in a large population of former psychiatric inpatients.	10.1111/j.1440-1819.2011.02205.x
Schneeweiss	2010	Variation in the Risk of Suicide Attempts and Completed Suicides by Antidepressant Agent in Adults: A Propensity Score-Adjusted Analysis of 9 Years' Data.	
Simon	2007	Suicide Attempts Among Patients Starting Depression Treatment With Medications or Psychotherapy	

Smith	2009	Association between consistent purchase of anticonvulsants or lithium and suicide risk: A longitudinal cohort study from Denmark, 1995-2001	https://dx.doi.org/10.1016/j.jad.2009.01.013
Sondergard	2006	Temporal changes in suicide rates for persons treated and not treated with antidepressants in Denmark during 1995-1999.	
Su	2019	Comparisons of the risk of medication noncompliance and suicidal behavior among patients with depressive disorders using different monotherapy antidepressants in Taiwan: A nationwide population-based retrospective cohort study	https://dx.doi.org/10.1016/j.jad.2019.03.039
Sung	2019	Concurrent use of benzodiazepines, antidepressants, and opioid analgesics with zolpidem and risk for suicide: a case-control and case-crossover study	https://dx.doi.org/10.1007/s00127-019-01713-x
Taipale	2024	Attention-Deficit/Hyperactivity Disorder Medications and Work Disability and Mental Health Outcomes	https://dx.doi.org/10.1001/jamanetworkopen.2024.2859
Tang	2022	Risks of all-cause death and completed suicide in patients with schizophrenia/schizoaffective disorder treated with long-acting injectable or oral antipsychotics: A population-based retrospective cohort study in Taiwan	https://dx.doi.org/10.1192/j.eurpsy.2021.2258
Tiihonen	2006	Effectiveness of antipsychotic treatments in a nationwide cohort of patients in community care after first hospitalisation due to schizophrenia and schizoaffective disorder: observational follow-up study.	10.1136/bmj.38881.382755.2F
Torrents	2020	Methadone poisonings: a seven-year retrospective study of the French poison center network focusing on suicide attempts vs. misuses	10.1111/fcp.12506

Ulcickas Yood	2010	Epidemiologic study of aripiprazole use and the incidence of suicide events	https://dx.doi.org/10.1002/pds.2047
Valuck	2009	Antidepressant discontinuation and risk of suicide attempt: A retrospective, nested case-control study	https://dx.doi.org/10.4088/JCP.08m04943
Van der Zalm	2021	Clozapine and mortality: A comparison with other antipsychotics in a nationwide Danish cohort study	https://dx.doi.org/10.1111/acps.13267
Vekaria	2024	Association of Past-Year Mental and Physical Health Conditions with Intentional or Unintentional Drug Overdoses	https://dx.doi.org/10.2139/ssrn.5041038
Walker	1997	Mortality in current and former users of clozapine.	
Wang	2024	Real-world evidence from a retrospective study on suicide during depression: clinical characteristics, treatment patterns and disease burden	https://dx.doi.org/10.1186/s12888-024-05726-y
Wang	2016	Comparison of suicide attempts/behaviors following smoking cessation treatments among schizophrenic smokers	
Ward	2006	Compliance with refilling prescriptions for atypical antipsychotic agents and its association with the risks for hospitalization, suicide, and death in patients with Schizophrenia in Quebec and Saskatchewan: A retrospective database study	https://dx.doi.org/10.1016/j.clinthera.2006.11.002
Wen	2010	Is antiepileptic drug use related to depression and suicidal ideation among patients with epilepsy?	https://dx.doi.org/10.1016/j.yebeh.2010.08.030
Wei	2022	Association of Long-Acting Injectable Antipsychotics and Oral Antipsychotics With Disease Relapse, Health Care Use, and Adverse Events Among People With Schizophrenia.	10.1001/jamanetworkopen.2022.24163

Werenberg	2018	Suicide risk in antiepileptic drug users - the role of previous suicidal behavior and psychiatric disease	https://dx.doi.org/10.1111/epi.14612
Wilkowska	2019	Clozapine: Promising treatment for suicidality in bipolar disorder.	
Wu	2017	Comparative risk of self-harm hospitalization amongst depressive disorder patients using different antidepressants: a population-based cohort study in Taiwan	https://dx.doi.org/10.1017/S0033291716002257
Yang	2024	Psychotropic Medications Promote Time-Dependent Reduction of Suicidal Ideation in Mood Disorder: A Prospective Cohort Study.	https://dx.doi.org/10.3346/jkms.2024.39.e226
Zheng	2024	Suicide-related risk among patients using branded and generic fluoxetine: a propensity score-matched, new-user design in Taiwan.	https://dx.doi.org/10.1186/s12888-024-06293-y
Zuo	2024	Medication non-adherence and self-inflicted violence behaviors among 185,800 patients with schizophrenia in the community: a 12-year cohort study.	https://dx.doi.org/10.1186/s12916-024-03354-7
**Outcome**			
Abrahamson	2017	Benzodiazepine, z-drug and pregabalin prescriptions and mortality among patients in opioid maintenance treatment-A nation-wide register-based open cohort study	https://dx.doi.org/10.1016/j.drugalcdep.2017.01.013
Akimoto	2016	Assessment of the risk of suicide-related events induced by concomitant use of antidepressants in cases of smoking cessation treatment with varenicline and assessment of latent risk by the use of varenicline	https://dx.doi.org/10.1371/journal.pone.0163583

Amendola	2024	Suicide rates and prescription of antidepressants: Trends in the United States, 1999-2020, by sex and race/ethnicity.	https://dx.doi.org/10.1027/0227-5910/a000941
Arendt	2013	Mortality following treatment for cannabis use disorders: Predictors and causes.	https://dx.doi.org/10.1016/j.jsat.2012.09.007
Azorin	2008	Evaluation of patients on sertindole treatment after failure of other antipsychotics: a retrospective analysis.	10.1186/1471-244X-8-16
Barak	2010	Suicidality and second generation antipsychotics in schizophrenia patients: A case-controlled retrospective study during a 5-year period.	https://dx.doi.org/10.1007/s00213-010-1843-6
Belsiyal	2022	Frequency of suicide ideation and attempts and its correlates among inpatients with depressive disorders at a tertiary care center in North India.	10.4103/jfmpc.jfmpc_2013_21
Bocchetta	2007	Long-term lithium treatment and survival from external causes including suicide.	https://dx.doi.org/10.1097/JCP.0b013e31814f4d94
Bogdanowicz	2016	Identifying mortality risks in patients with opioid use disorder using brief screening assessment: Secondary mental health clinical records analysis	10.1016/j.drugalcdep.2016.04.036
Bogdanowicz	2015	Double trouble: Psychiatric comorbidity and opioid addiction-All-cause and cause-specific mortality	10.1016/j.drugalcdep.2014.12.025
Brancati	2023	Differential characteristics of bipolar I and II disorders: a retrospective, cross-sectional evaluation of clinical features, illness course, and response to treatment	https://dx.doi.org/10.1186/s40345-023-00304-9
Brenner	2021	Excess deaths in treatment-resistant depression	https://dx.doi.org/10.1177/20451253211006508

Britton	2010	Suicide Attempts within 12 Months of Treatment for Substance Use Disorders.	10.1521/suli.2010.40.1.14
Buggy	2013	Neuropsychiatric events with varenicline: A modified prescription-event monitoring study in general practice in England	https://dx.doi.org/10.1007/s40264-013-0046-6
Chan	2022	Longitudinal relapse pattern of patients with first-episode schizophrenia-spectrum disorders and its predictors and outcomes: A 10-year follow-up study	https://dx.doi.org/10.1016/j.ajp.2022.103087
Clapham	2022	Suicide in Schizophrenia and Adverse Events During Antipsychotic Medication	https://www.diva-portal.org/smash/get/diva2:1700500/FULLTEXT01.pdf
Colman	2008	Factors associated with antidepressant, anxiolytic and hypnotic use over 17 years in a national cohort	https://dx.doi.org/10.1016/j.jad.2008.01.021
Coppen	1998	Suicide mortality in patients on lithium maintenance therapy	https://dx.doi.org/10.1016/S0165-0327%2898%2900067-6
Coryell	2003	The Long-term Course of Rapid-Cycling Bipolar Disorder.	
Darke	2001	The relationship between suicide and heroin overdose among methadone maintenance patients in Sydney, Australia.	
Davies	2009	Interim results of a modified prescription event monitoring study on atomoxetine; reports of suicide related behaviour	https://dx.doi.org/10.1002/pds.1806
Demir	2022	Substance use characteristics, treatment completion rates and related factors of patients in Gaziantep AMATEM in 2019; a retrospective study.	https://dx.doi.org/10.1080/14659891.2021.1912202
Ding	2024	Trajectories and predictors of suicidal ideation in clinical characteristics and pharmacological treatments for major	https://dx.doi.org/10.1038/s41398-024-03115-3

		depressive disorder: a study based on a national multi-centered prospective cohort.	
Dupuoy	2014	Association between benzodiazepine drugs and total mortality: Evidence from a study in the EGB	https://dx.doi.org/10.1111/fcp.12064 PT
Edlinger	2018	Trends in pharmacological emergency treatment of patients suffering from schizophrenia over a 16-year observation period.	https://dx.doi.org/10.1097/YIC.0000000000000220
Fagot	2016	Cohort of one million patients initiating antidepressant treatment in France: 12-month follow-up	https://dx.doi.org/10.1111/ijcp.12850
Fazel	2006	Suicide trends in discharged patients with mood disorders: associations with selective serotonin uptake inhibitors and comorbid substance misuse.	
Fond	2023	Long-term benzodiazepine prescription in treatment-resistant depression: A national FACE-TRD prospective study	https://dx.doi.org/10.1016/j.pnpbp.2023.110779
Fontanella	2016	Benzodiazepine Use and Risk of Mortality Among Patients With Schizophrenia: A Retrospective Longitudinal Study	10.4088/JCP.15m10271
Fudalej	2015	Somatic Comorbidity and Other Factors Related to Suicide Attempt Among Polish Methadone Maintenance Patients.	10.1097/ADM.0000000000000153
Gaertner	2002	A case control study on psychopharmacotherapy before suicide committed by 61 psychiatric inpatients	https://dx.doi.org/10.1055/s-2002-25027
Gao	2016	Risk-factors for methadone-specific deaths in Scotland's methadone-prescription clients between 2009 and 2013	10.1016/j.drugalcdep.2016.08.627

Gasquet	2009	The European Schizophrenia Outpatient Health Outcomes Study: Effectiveness results of the French cohort of schizophrenic outpatients over a 36-month follow-up	https://dx.doi.org/10.1016/j.respe.2008.11.001
Geith	2022	Lessons to be learned: identifying high-risk medication and circumstances in patients at risk for suicidal self-poisoning	https://dx.doi.org/10.1186/s13033-021-00513-8
Gibbons	2005	The relationship between antidepressant medication use and rate of suicide	https://dx.doi.org/10.1001/archpsyc.62.2.165
Gorwood	2016	Efficacy and safety of agomelatine over one year. A French prospective observational study of depressed patients followed-up in medical practice	
Gossop	2002	A prospective study of mortality among drug misusers during a 4-year period after seeking treatment	10.1046/j.1360-0443.2002.00079.x
Guaiana	2011	Sales of antidepressants, suicides and hospital admissions for depression in Veneto Region, Italy, from 2000 to 2005: An ecological study.	https://dx.doi.org/10.1186/1744-859X-10-24
Gundogmus	2021	Clinical and demographic factors associated with early relapse in patients with schizophrenia: A naturalistic observation study	https://dx.doi.org/10.1097/YIC.0000000000000377
Gunnell	2009	Varenicline and suicidal behaviour: A cohort study based on data from the General Practice Research Database.	
Hallvik	2022	Patient outcomes after opioid dose reduction among patients with chronic opioid therapy	10.1097/j.pain.0000000000002298
Haw	2002	Deliberate self harm patients with depressive disorders: Treatment and outcome.	https://dx.doi.org/10.1016/S0165-0327%2801%2900317-2

Hedstrom	2020	Short- and long-term mortality following hypnotic use	10.1111/jsr.13061
Ho	2017	Major depression and related factors among heroin users who received methadone maintenance treatment in Taiwan: a case control study	https://dx.doi.org/10.3109/14659891.2015.1021866
Huang	2013	Factors associated with mortality among heroin users after seeking treatment with methadone: A population-based cohort study in Taiwan	https://dx.doi.org/10.1016/j.jsat.2012.08.003
Huang	2021	Clinical characteristics and rehospitalization in patients with schizophrenia with or without history of amphetamine abuse.	10.4103/TPSY.TPSY_5_21
Janzen	2022	Second-Generation Long-Acting Injectable Antipsychotics and the Risk of Treatment Failure in a Population-Based Cohort	https://dx.doi.org/10.3389/fphar.2022.879224
Josephson	2018	Prescription trends and psychiatric symptoms following first receipt of one of seven common antiepileptic drugs in general practice	10.1016/j.yebeh.2018.04.012
Kelty	2018	Self-injuring behavior and mental illness in opioid-dependent patients treated with implant naltrexone, methadone, and buprenorphine in Western Australia.	https://dx.doi.org/10.1007/s11469-017-9856-6
Kendall	2017	A cohort study examining emergency department visits and hospital admissions among people who use drugs in Ottawa, Canada	https://dx.doi.org/10.1186/s12954-017-0143-4
Kermode	2020	Retention and outcomes for clients attending a methadone clinic in a resource-constrained setting: A mixed methods prospective cohort study in Imphal, Northeast India	https://dx.doi.org/10.1186/s12954-020-00413-z

Kern	2021	Treatment patterns of patients diagnosed with major depressive disorder and suicidal ideation or attempt: A U.S. population-based study utilizing real-world data.	https://dx.doi.org/10.1186/s12888-021-03616-1
Kessing	2011	Valproate V. lithium in the treatment of bipolar disorder in clinical practice: observational nationwide register-based cohort study	10.1192/bjp.bp.110.084822
Ketcham	2024	Longitudinal study of insomnia, suicidal ideation, and psychopathology in schizophrenia	https://dx.doi.org/10.1016/j.schres.2024.03.030
Klovgaard	2021	Sudden unexpected death in epilepsy in persons younger than 50 years: A retrospective nationwide cohort study in Denmark	10.1111/epi.17037
Krivoy	2011	Predictors of clozapine discontinuation in patients with schizophrenia.	10.1097/YIC.0b013e32834ab34c
Kulkarni	2009	Two-year treatment outcomes of an Australian outpatient cohort with bipolar I or schizoaffective disorder	https://dx.doi.org/10.1111/j.1399-5618.2009.00751.x
Lam	2022	Pharmaceutical opioid poisonings in Victoria, Australia: Rates and characteristics of a decade of emergency department presentations among nine pharmaceutical opioids	https://dx.doi.org/10.1111/add.15653
Lee	2023	Long-Term Outcome of Clozapine in Treatment-Resistant Schizophrenia.	10.1097/JCP.0000000000001671
Lekka	2002	Suicide attempts in high-dose benzodiazepine users.	
Licht	2008	Long-term outcome of patients with bipolar disorder commenced on lithium prophylaxis during hospitalization: A complete 15-year register-based follow-up	https://dx.doi.org/10.1111/j.1399-5618.2008.00499.x

Lundberg	2023	Association of Treatment-Resistant Depression With Patient Outcomes and Health Care Resource Utilization in a Population-Wide Study	https://dx.doi.org/10.1001/jamapsychiatry.2022.3860
Madadi	2013	New risk factors for opioid-related deaths in ontarians	
Maria Pavarin	2008	Mortality risk in intravenous drug users in Bologna and its determining factors. Results of a longitudinal study	
Maxwell	2005	Deaths of clients in methadone treatment in Texas: 1994-2002	https://dx.doi.org/10.1016/j.drugalcdep.2004.09.006
Mines	2005	Prevalence of risk factors for suicide in patients prescribed venlafaxine, fluoxetine, and citalopram	https://dx.doi.org/10.1002/pds.1095
Miotto	1997	Overdose, suicide attempts and death among a cohort of naltrexone-treated opioid addicts	https://dx.doi.org/10.1016/S0376-8716%2897%2901348-3
Molero	2015	Varenicline and risk of psychiatric conditions, suicidal behaviour, criminal offending, and transport accidents and offences: population based cohort study.	
Moore	2011	Suicidal behavior and depression in smoking cessation treatments.	https://dx.doi.org/10.1371/journal.pone.0027016
Mora	2024	Lurasidone uses and dosages in Spain: RETROLUR, a real-world retrospective analysis using artificial intelligence.	https://dx.doi.org/10.3389/fpsyt.2024.1506142
MullerOerlinghausen	1996	Mortality of patients who dropped out from regular lithium prophylaxis: A collaborative study by the International Group for the Study of Lithium-Treated Patients (IGSLI)	10.1111/j.1600-0447.1996.tb09870.x
Musetti	2022	Which patients with bipolar depression receive antidepressant augmentation? Results from an observational multicenter study	https://dx.doi.org/10.1017/S109285292100078X

Nelson	2017	Morbidity and mortality associated with medications used in the treatment of depression: An analysis of cases reported to U.S. Poison Control Centers, 2000-2014.	https://dx.doi.org/10.1176/appi.ajp.2016.16050523
Nevalainen	2013	Long-term mortality risk by cause of death in newly diagnosed patients with epilepsy in Finland: A nationwide register-based study	https://dx.doi.org/10.1007/s10654-013-9848-1
Ng-Mak	2019	Hospitalization risk in bipolar disorder patients treated with lurasidone versus other atypical antipsychotics	10.1080/03007995.2018.1462787
Novick	2006	Remission and relapse in the outpatient care of schizophrenia: Three-year results from the schizophrenia outpatient health outcomes study	https://dx.doi.org/10.1097/01.jcp.0000246215.49271.b8
Nyhlen	2011	Causes of premature mortality in Swedish drug abusers: A prospective longitudinal study 1970-2006	https://dx.doi.org/10.1016/j.jflm.2011.01.003
Okumura	2017	Risk of recurrent overdose associated with prescribing patterns of psychotropic medications after nonfatal overdose	10.2147/NDT.S128278
Okumura	2015	Exposure to psychotropic medications prior to overdose: a case-control study	10.1007/s00213-015-3952-8
Pan	2024	Psychotropic medications and mortality from cardiovascular disease and suicide for individuals with depression in Taiwan	https://dx.doi.org/10.1016/j.ajp.2024.104091
Patorno	2010	Anticonvulsant medications and the risk of suicide, attempted suicide, or violent death.	https://dx.doi.org/10.1001/jama.2010.410
Patorno	2017	Benzodiazepines and risk of all cause mortality in adults: cohort study	10.1136/bmj.j2941

Payne	2009	Patterns and predictors of re-admission to hospital with self-poisoning in Scotland	10.1016/j.puhe.2008.12.002
Poluzzi	2013	Trend in SSRI-SNRI antidepressants prescription over a 6-year period and predictors of poor adherence	10.1007/s00228-013-1567-8
Pompili	2023	Lithium treatment versus hospitalization in bipolar disorder and major depression patients.	https://dx.doi.org/10.1016/j.jad.2023.08.028
Portela	2020	Antipsychotics used to treat patients with severe mental disorder in outpatient follow-up at a psychiatric hospital	https://dx.doi.org/10.1002/pds.5114
Raja	2009	Psychopharmacological treatment before suicide attempt among patients admitted to a psychiatric intensive care unit.	https://dx.doi.org/10.1016/j.jad.2008.04.024
Reece	2010	Favorable Mortality Profile of Naltrexone Implants for Opiate Addiction	10.1080/10550880903435988
Reseland	2008	National Suicide Rates 1961-2003: Further Analysis of Nordic Data for Suicide, Autopsies and Ill-Defined Death Rates.	
Reutfors	2018	Mortality in treatment-resistant unipolar depression: A register-based cohort study in Sweden	https://dx.doi.org/10.1016/j.jad.2018.06.030
Rose	2020	Causes of death in clozapine-treated patients in a catchment area: a 10-year retrospective case-control study	https://dx.doi.org/10.1016/j.euroneuro.2020.05.011
Roy	2021	Disease prevalence, comorbidities, and medication utilization among patients with opioid use disorder in the US	https://dx.doi.org/10.1002/pds.5305
Schifano	2012	Impact of an 18-month, NHS-based, treatment exposure for heroin dependence: Results from the London area treat 2000 study.	https://dx.doi.org/10.1111/j.1521-0391.2012.00226.x

Seemuller	2014	Three-year long-term outcome of 458 naturalistically treated inpatients with major depressive episode: Severe relapse rates and risk factors.	https://dx.doi.org/10.1007/s00406-014-0495-7
Shuy	2024	Pharmacoepidemiology and Clinical Correlates of Lithium Treatment for Bipolar Disorder in Asia.	10.1097/JCP.0000000000001813
Sood	2000	Determinants of antidepressant treatment outcome	
Soyka	2006	One-year mortality rates of patients receiving methadone and buprenorphine maintenance therapy: A nationally representative cohort study in 2694 patients	https://dx.doi.org/10.1097/01.jcp.0000245561.99036.49
Stiltner	2023	Polysubstance addiction and psychiatric, somatic comorbidities among 7,989 individuals with cocaine use disorder: a latent class analysis	https://dx.doi.org/10.1101/2023.02.08.23285653
Stroup	2016	Comparative Effectiveness of Clozapine and Standard Antipsychotic Treatment in Adults With Schizophrenia	10.1176/appi.ajp.2015.15030332
Sundell	2011	Antidepressant utilization patterns and mortality in Swedish men and women aged 20-34 years	10.1007/s00228-010-0933-z
Szmulewicz	2023	Emulating a Target Trial of Dynamic Treatment Strategies for Major Depressive Disorder Using Data From the STAR*D Randomized Trial	https://dx.doi.org/10.1016/j.biopsych.2022.09.028
Tadrous	2016	Association of varenicline compared to bupropion and self-harm.	10.1371/journal.pone.0163681.t002
Tadrous	2016	Varenicline and risk of self-harm: A nested case-control study	https://dx.doi.org/10.1002/pds.4070
Taipale	2024	Real-world effectiveness of antidepressants, antipsychotics and their combinations in the maintenance treatment of psychotic	https://dx.doi.org/10.1002/wps.21205

		depression. Evidence from within-subject analyses of two nationwide cohorts.	
Tait	2008	Mortality in heroin users 3 years after naltrexone implant or methadone maintenance treatment	https://dx.doi.org/10.1016/j.jsat.2007.08.014
Tenback	2012	All-Cause Mortality and Medication Risk Factors in Schizophrenia A Prospective Cohort Study	10.1097/JCP.0b013e31823f3c43
Thomas	2013	Smoking cessation treatment and risk of depression, suicide, and self harm in the Clinical Practice Research Datalink: Prospective cohort study	https://dx.doi.org/10.1136/bmj.f5704
Tiihonen	2017	Real-world effectiveness of antipsychotic treatments in a nationwide cohort of 29, 823 patients	
Tournier	2019	Conventional mood stabilizers and/or second-generation antipsychotic drugs in bipolar disorders: A population-based comparison of risk of treatment failure	https://dx.doi.org/10.1016/j.jad.2019.07.054
Troberg	2023	Malmo Treatment Referral and Intervention Study (MATRIS)-36-month follow-up on retention and substance use among patients referred from needle exchange to opioid agonist treatment-The role of stimulant use at baseline.	https://dx.doi.org/10.1016/j.josat.2023.209036
Tundo	2021	Is short-term antidepressant treatment effective and safe in bipolar depression? Results from an observational multicenter study	https://dx.doi.org/10.1002/hup.2773
Uosukainen	2013	Mortality among clients seeking treatment for buprenorphine abuse in Finland	10.1016/j.drugalcdep.2013.06.022

Vanasse	2016	Comparative effectiveness and safety of antipsychotic drugs in schizophrenia treatment: A real-world observational study.	https://dx.doi.org/10.1111/acps.12621
Vieta	2008	A long-term prospective study on the outcome of bipolar patients treated with long-acting injectable risperidone	10.1080/15622970701530917
von Oelreich	2024	Antidepressant drug use after intensive care: a nationwide cohort study.	https://dx.doi.org/10.1038/s41598-024-66028-7
Yin	2024	History of suicidal behavior and clozapine prescribing among people with schizophrenia in China: a cohort study.	https://dx.doi.org/10.1186/s12888-024-05893-y
Zagozdzon	2015	Mortality in users of conventional vs. Atypical antipsychotic medications in Poland	
Zoltan	2015	Treatment of bipolar depression with lamotrigine - rate of relapse and suicidal behaviour during 6 month follow-up treatment	http://www.mppt.hu/images/magazin/pdf/xvii-evfolyam-1-szam/rihmer.pdf
**Intervention**			
Angst	2002	Mortality of patients with mood disorders: Follow-up over 34-38 years.	https://dx.doi.org/10.1016/S0165-0327%2801%2900377-9
Artenie	2015	Licit and illicit substance use among people who inject drugs and the association with subsequent suicidal attempt.	https://dx.doi.org/10.1111/add.13030
Balestrieri	2022	Suicide risk in medically ill inpatients referred to consultation-liaison psychiatric services: A multicenter study	https://dx.doi.org/10.1016/j.jad.2022.08.113
Baltazar	2022	Long term course and outcome of first episode schizophrenia: a 27-to-31-year follow-up	https://dx.doi.org/10.1007/s00127-021-02185-8

Beautrais	2006	Suicidal behaviour in Te Rau Hinengaro: The New Zealand Mental Health Survey.	
Breier	1991	National Institute of Mental Health longitudinal study of chronic schizophrenia. Prognosis and predictors of outcome	
Brenner	2019	Risk factors for suicide and suicide attempts among patients with treatment resistant depression: A population based nested case-control study	https://dx.doi.org/10.1002/pds.4864
Brenner	2020	Excess deaths associated with treatment resistance in patients with major depressive disorder	https://dx.doi.org/10.1002/pds.5114
Brugnoli	2012	Risk factors for suicide behaviors in the observational schizophrenia outpatient health outcomes (SOHO) study	10.1186/1471-244X-12-83
Chang	2010	All-cause mortality among people with serious mental illness (SMI), substance use disorders, and depressive disorders in southeast London: a cohort study	10.1186/1471-244X-10-77
Chang	2012	Survival of bipolar depression, other type of depression and comorbid ailments: Ten-year longitudinal follow-up of 10,922 Taiwanese patients with depressive disorders (KCIS no. PSY1)	10.1016/j.jpsychires.2012.07.014
Chang	2017	Causes of death and expected years of life lost among treated opioid-dependent individuals in the United States and Taiwan	10.1016/j.drugpo.2016.12.003
Chartrand	2012	A longitudinal population-based study exploring treatment utilization and suicidal ideation and behavior in major depressive disorder.	https://dx.doi.org/10.1016/j.jad.2012.03.040

Chen	2014	Suicide risk in major affective disorder: Results from a national survey in China.	https://dx.doi.org/10.1016/j.jad.2013.10.046
Cho	2020	Temporal association between zolpidem medication and the risk of suicide: A 12-year population-based, retrospective cohort study	10.1038/s41598-020-61694-9
Choi	2018	Zolpidem use and suicide death in South Korea: A population-based case-control study	https://dx.doi.org/10.1002/pds.4629
Crump	2021	Comparative risk of suicide by specific substance use disorders: A national cohort study	https://dx.doi.org/10.1016/j.jpsychires.2021.10.017
Demidenko	2017	Suicidal ideation and suicidal self-directed violence following clinician-initiated prescription opioid discontinuation among long-term opioid users	https://dx.doi.org/10.1016/j.genhosppsych.2017.04.011
Dutta	2007	Suicide and other causes of mortality in bipolar disorder: a longitudinal study	10.1017/S0033291707000347
Fournier	2018	Association between binge drug use and suicide attempt among people who inject drugs.	https://dx.doi.org/10.1080/08897077.2017.1389800
Gibbons	2023	Benztropine and suicide attempts and intentional self-harm	https://dx.doi.org/10.1016/j.psychres.2023.115054
Gibbons	2021	Concomitant opioid and benzodiazepine use and risk of suicide attempt and intentional self-harm: Pharmacoepidemiologic study.	https://dx.doi.org/10.1016/j.drugalcdep.2021.109046
Gonzalez-Pinto	2007	Predictors of suicide in first-episode affective and nonaffective psychotic inpatients: Five-year follow-up of patients from a catchment area in Vitoria, Spain	https://dx.doi.org/10.4088/JCP.v68n0209
Gorton	2018	Risk of unnatural mortality in people with epilepsy	https://dx.doi.org/10.1001/jamaneurol.2018.0333

Gorton	2018	Self-harm in people with epilepsy: Population-based cohort and nested casecontrol studies	https://dx.doi.org/10.1002/pds.4629
Gorton	2018	Risk factors for self-harm in people with epilepsy	https://dx.doi.org/10.1007/s00415-018-9094-2
Gronemann	2020	All-cause mortality, suicide and self-harm in patients with treatment-resistant depression	https://dx.doi.org/10.1002/pds.5114
Gronemann	2021	Treatment-resistant depression and risk of all-cause mortality and suicidality in Danish patients with major depression.	https://dx.doi.org/10.1016/j.jpsychires.2021.01.014
Hawton	2007	Self-harm in England: A tale of three cities: Multicentre study of self-harm.	https://dx.doi.org/10.1007/s00127-007-0199-7
Healy	2006	Lifetime suicide rates in treated schizophrenia: 1875-1924 and 1994-1998 cohorts compared +.	
Houston	2003	General practitioner contacts with patients before and after deliberate self harm	
Jiang	2021	Suicide and non-fatal suicide attempts among persons with depression in the population of Denmark.	
Kohler-Forsberg	2020	The effect of combined treatment with SSRIs and renin-angiotensin system (RAS) drugs: A propensity score matched cohort study	https://dx.doi.org/10.1016/j.euroneuro.2020.01.004
Kuo	2011	Risk and Protective Factors for Suicide Among Patients With Methamphetamine Dependence: A Nested Case-Control Study	10.4088/JCP.09m05360gry
Kurdyak	2021	Mortality After the First Diagnosis of Schizophrenia-Spectrum Disorders: A Population-based Retrospective Cohort Study	10.1093/schbul/sbaa180

Lee	2013	Suicide and other-cause mortality among heroin users in Taiwan: A prospective study	https://dx.doi.org/10.1016/j.addbeh.2013.03.003
Lee	2021	All-cause and suicide mortality among people with methamphetamine use disorder: a nation-wide cohort study in Taiwan	10.1111/add.15501
Lee	2022	Healthcare utilization and psychiatric and physical comorbidities before suicide mortality in patients with methamphetamine use disorder: A nationwide case-control study	10.1016/j.addbeh.2021.107192
Liang	2018	Relationship between mortality in people with mental disorders and suicide mortality in China during 2000 to 2014 An observational study	10.1097/MD.0000000000013359
Li	2023	Factors Influencing Suicide Deaths in Patients With Schizophrenia Based on Cohort Data: An Empirical Study of a Sample of 170006 Patients in Sichuan Province	https://dx.doi.org/10.12182/20230160302 PT - Article
Madsen	2021	Cause-specific life years lost in individuals with treatment-resistant depression: A Danish nationwide register-based cohort study	https://dx.doi.org/10.1016/j.jad.2020.11.042
Merrall	2012	Mortality of those who attended drug services in Scotland 1996-2006: Record-linkage study	10.1016/j.drugpo.2011.05.010
Meyer	2014	Self-harm in people with epilepsy: A retrospective cohort study	https://dx.doi.org/10.1111/epi.12723
Molero	2023	Associations between β-blockers and psychiatric and behavioural outcomes: A population-based cohort study of 1.4 million individuals in Sweden	10.1371/journal.pmed.1004164

Niederkrotenthaler	2020	Healthcare utilization, psychiatric medication and risk of rehospitalization in suicide-attempting patients with common mental disorders.	https://dx.doi.org/10.1177/0004867419895112
Osborn	2008	Suicide and severe mental illnesses. Cohort study within the UK general practice research database	https://dx.doi.org/10.1016/j.schres.2007.11.025
Oyefeso	1999	Suicide among drug addicts in the UK.	
Pan	2014	Excessive suicide mortality and risk factors for suicide among patients with heroin dependence	10.1016/j.drugalcdep.2014.10.021
Pavarin	2023	Epidemiology and Clinical-Demographic Characteristics of Suicide Attempts in Alcohol Use Disorders in an Italian Population	https://dx.doi.org/10.1080/02791072.2022.2107464
Penttinen	2001	Risk of suicide and accidental death among subjects visiting a doctor because of mental disorder: A matched case-control study in Finnish farmers	10.1539/joh.43.107
Plans	2019	Completed suicide in bipolar disorder patients: A cohort study after first hospitalization	https://dx.doi.org/10.1016/j.jad.2019.07.048
Porras-Segovia	2023	Factors associated with transitioning from suicidal ideation to suicide attempt in the short-term: Two large cohorts of depressed outpatients	10.1016/j.jad.2023.05.018
Qin	2006	Trends in suicide risk associated with hospitalized psychiatric illness: A case-control study based on Danish longitudinal registers	10.4088/JCP.v67n1214
Reutfors	2021	Risk Factors for Suicide and Suicide Attempts Among Patients With Treatment-Resistant Depression: Nested Case-Control Study	10.1080/13811118.2019.1691692

Reutfors	2016	Suicide risk and antipsychotic side effects in schizophrenia: nested case-control study	10.1002/hup.2536
Sherry	2023	A National Retrospective Study of Antidepressants' Effects on Overdose and Self-Harm Among Adults Treated With Opioid Analgesics	https://dx.doi.org/10.1176/appi.ps.20220070
Stromme	2024	Risk factors for mortality in patients admitted to a psychiatric acute ward: A prospective cohort study	10.1111/acps.13657
Vold	2022	Prevalence and correlates of suicide attempts in high-risk populations: a cross-sectional study among patients receiving opioid agonist therapy in Norway	https://dx.doi.org/10.1186/s12888-022-03829-y
von Greiff	2018	Mortality and Cause of DeathA 30-Year Follow-Up of Substance Misusers in Sweden	10.1080/10826084.2018.1452261
Xin	2021	Relationship between suicide rate and antidepressant prescription: An ecological study in the People's Republic of China.	https://dx.doi.org/10.1002/hup.2760
**Population**			
Beckman	2016	Mental illness and suicide after self-harm among young adults: Long-term follow-up of self-harm patients, admitted to hospital care, in a national cohort.	https://dx.doi.org/10.1017/S0033291716002282
Chen	2020	Suicide attempts and death among heroin-involved women seeking methadone treatment in Taiwan	https://dx.doi.org/10.1016/j.drugalcdep.2020.108277
Gottlieb	2023	A comparison of mortality rates for buprenorphine versus methadone treatments for opioid use disorder	https://dx.doi.org/10.1111/acps.13477

Hamina	2024	Fatal drug overdoses in individuals treated pharmacologically for chronic pain: a nationwide register-based study	https://dx.doi.org/10.1016/j.bja.2023.10.016
Larney	2014	Opioid substitution therapy as a strategy to reduce deaths in prison: retrospective cohort study	10.1136/bmjopen-2013-004666
Lee	2018	Clinical epidemiology of long-term suicide risk in a nationwide population-based cohort study in South Korea	https://dx.doi.org/10.1016/j.jpsychires.2018.01.018
Linden	2013	Risk of suicide and suicide attempt associated with atomoxetine compared to central nervous system stimulant treatment	https://dx.doi.org/10.1002/pds.3512
Man	2017	Association of Risk of Suicide Attempts With Methylphenidate Treatment.	10.1001/jamapsychiatry.2017.2183
McCarthy	2009	Mortality associated with attention-deficit hyperactivity disorder (ADHD) drug treatment: A retrospective cohort study of children, adolescents and young adults using the general practice research database	https://dx.doi.org/10.2165/11317630-000000000-00000
Rohde	2018	Real-world effectiveness of clozapine for intellectual disability: Results from a mirror-image and a reverse-mirror-image study.	10.1177/0269881118783322
Rush	2024	A framework for inferring and analyzing pharmacotherapy treatment patterns.	https://dx.doi.org/10.1186/s12911-024-02469-4
Sondergard	2006	Do antidepressants prevent suicide?	https://dx.doi.org/10.1097/00004850-200607000-00003
Sorensen	2001	Risk of suicide in users of β-adrenoceptor blockers, calcium channel blockers and angiotensin converting enzyme inhibitors	10.1046/j.0306-5251.2001.01442.x

Stralin	2019	Medication, hospitalizations and mortality in 5 years after first-episode psychosis in a Swedish nation-wide cohort	https://dx.doi.org/10.1111/eip.12697
Teferra	2011	Five-year mortality in a cohort of people with schizophrenia in Ethiopia.	https://dx.doi.org/10.1186/1471-244X-11-165
Thomas	2024	Suicide, Stimulants, and Selective Serotonin Reuptake Inhibitors: A Retrospective Chart Review.	https://dx.doi.org/10.1089/cap.2023.0068
Wimberley	2017	Mortality and self-harm in association with clozapine in treatment-resistant schizophrenia	https://dx.doi.org/10.1176/appi.ajp.2017.16091097
Woody	2007	Premature Deaths After Discharge from Methadone Maintenance: A Replication.	10.1097/ADM.0b013e318155980e
**Duplicated samples and other background papers**			
Andersohn	2009	Use of antiepileptic drugs in epilepsy and the risk of self harm and suicidal behaviour	https://dx.doi.org/10.1002/pds.1806
Bralet	2000	[Cause of mortality in schizophrenic patients: prospective study of years of a cohort of 150 chronic schizophrenic patients].	
Brodersen	2001	Mortality in patients with affective disorder who commenced treatment with lithium. A 16-year follow-up	
Chang	2015	Estimation of life expectancy and the expected years of life lost among heroin users in the era of opioid substitution treatment (OST) in Taiwan	10.1016/j.drugalcdep.2015.05.033
Fernandez Miranda	2019	Suicide prevention with second-generation long-acting-injectable antipsychotics among people with severe schizophrenia	https://doi.org/10.1080/24750573.2019.1603432

Fernandez-Miranda	2021	Oral versus long-acting injectable antipsychotic treatment for people with severe schizophrenia: A 5-year follow-up of effectiveness.	https://dx.doi.org/10.1097/NMD.0000000000001299
Haukka	2006	Antidepressants and the risk of suicide, attempted suicide and overall mortality in a nation-wide cohort	
Katz	2018	Suicide risk in bipolar disorder: Comparing lithium, divalproex, and carbamazepine.	
Kelty	2012	Examination of mortality rates in a retrospective cohort of patients treated with oral or implant naltrexone for problematic opiate use	10.1111/j.1360-0443.2012.03910.x
Rahman	2022	Disability pension due to common mental disorders: Subsequent psychiatric morbidity and suicidal behaviour.	
Ruengorn	2011	Incidence and risk factors of suicide reattempts within 1 year after psychiatric hospital discharge in mood disorder patients	https://www.dovepress.com/getfile.php?fileID=11435
Song	2017	Suicidal behavior during lithium and valproate treatment: A within-individual 8-year prospective study of 50,000 patients with bipolar disorder.	https://dx.doi.org/10.1176/appi.ajp.2017.16050542
Sun	2015	Zolpidem and the risk of suicide: A nationwide population-based case-control study	https://dx.doi.org/10.1016/j.jns.2015.08.649
Tiihonen	2009	11-year follow-up of mortality in patients with schizophrenia: a population-based cohort study (FIN11 study)	10.1016/S0140-6736(09)60742-X
Tondo	2024	Prevention of suicidal behavior with lithium treatment in patients with recurrent mood disorders.	https://dx.doi.org/10.1186/s40345-024-00326-x

Tournier	2022	Risk of suicidal behavior associated with benzodiazepines: A nationwide case-crossover study	
Tournier	2016	Outcomes of three treatment strategies in bipolar disorder using conventional mood stabilizers and antipsychotic drugs	https://dx.doi.org/10.1002/pds.4070
Valuck	2005	Antidepressant treatment and risk of suicide attempt by adults with major depressive disorder: A propensity-adjusted retrospective cohort study	
Yager	2016	Do Statins Improve Antidepressant Effectiveness?	10.1056/nejm-jw.NA41161
Ziemba	2010	Do Antiepileptic Drugs Increase the Risk of Suicidality in Adult Patients With Epilepsy?: A Critically Appraised Topic.	10.1097/NRL.0b013e3181f79f37
**Publication type (conference abstracts, research proposals, etc.)**			
Abdullah	2010	National mental health registry - Schizophrenia one year outcome study	http://www.e-mjm.org/2010/CRC_2010_supA.pdf
Arana	2011	"Suicide-related Events in Patients Treated With Antiepileptic Drugs"	10.1097/EDE.0b013e31823198fc
Astrup	2013	Impact of censoring at or truncating risk time of hospitalizations on the association between antiepileptic drugs and suicide attempt	https://dx.doi.org/10.1007/s10654-013-9820-0
Barbui	2014	Antidepressant dose and the risk of deliberate self-harm	https://dx.doi.org/10.1017/S2045796014000456
Bellivier	2013	Wave-BD, an ambispective multinational observational study on bipolar I and II disorder (BDI, BDII): French cohort clinical outcomes (nct01062607)	

Berardelli	2024	The role of long-acting antipsychotics in illness relapse: an observational study	https://dx.doi.org/10.1192/j.eurpsy.2024.805
Bjorkholm	2021	P.0871 Epidemiology of major depression and intentional self-harm from a total-population cohort in the greater Stockholm region	https://dx.doi.org/10.1016/j.euroneuro.2021.10.727
Cano	2015	Clinical outcomes in schizophrenia patients during first years of diagnosis, a cohort study	https://dx.doi.org/10.1017/S1092852914000765
Chang	2022	Use of central nervous system drugs in combination with selective serotonin reuptake treatment: A Bayesian screening study for risk of suicidal behavior	https://dx.doi.org/10.1002/pds.5518
Coon	2022	Risk for Suicidal Ideation with Atomoxetine And Bupropion In Attention-Deficit/Hyperactivity Disorder: A Cohort Study	https://dx.doi.org/10.1002/jac5.1661
Crocq	2010	Suicide attempts in the SCoP study	https://dx.doi.org/10.1016/j.schres.2010.02.692
De Hert	2009	"Mortality in patients with schizophrenia": Comment.	https://dx.doi.org/10.1016/S0140-6736%2809%2961943-7
Denee	2022	POSA152 Characterising Patients with Major Depressive Disorder with Moderate-to-High Suicide Intent and Their Healthcare Resource Utilisation in England	https://dx.doi.org/10.1016/j.jval.2021.11.293
Diaz-Fernandez	2018	Clinical and rehabilitation treatment outcomes of patients with severe schizophrenia in a comprehensive, case managed programme. A 7-year follow-up	https://dx.doi.org/10.1016/j.eurpsy.2017.12.022
Dubovsky	2016	Lithium: Still the One.	10.1056/nejm-jw.NA41376

Fernandez Miranda	2015	Treatment outcomes of patients with severe schizophrenia undergoing specific severe mental illness programme. A 6-year follow-up	
Fructuoso	2014	Suicide attempt and medications. A retrospective case-control study	https://dx.doi.org/10.1111/bcpt.12301
Garcia-Carmona	2018	Paliperidone long-acting injectable (LAI) is associated with a lower intake of benzodiazepines and a lower number of admissions compared with other lais in a cohort of patients with schizophrenia	https://dx.doi.org/10.1093/schbul/sby016.507
Gasse	2013	Impact of previous suicide attempts and family history of psychiatric disease on the size of the association between antiepileptic drugs and suicide related events	https://dx.doi.org/10.1002/pds.3512
Grabner	2024	HSD70 Characteristics of Medicare Patients Initiating Long-Acting Injectable Antipsychotic Medications	https://dx.doi.org/10.1016/j.jval.2024.03.1293
Granbichler	2012	Causes of death in epilepsy patients	
Granbichler	2011	Cause-specific mortality among patients with epilepsy: Results of a 40-year cohort study	https://dx.doi.org/10.1111/j.1528-1167.2011.03206.x
Hamina	2022	Fatal overdoses in a cohort of chronic pain patients	https://dx.doi.org/10.1002/pds.5518
Hoier	2022	The association between benzodiazepine and nonbenzodiazepine and suicide: a nationwide cohort study	https://dx.doi.org/10.1192/j.eurpsy.2022.478
Hopkins	1998	Duration of untreated psychosis and outcome in a 10 year follow-up cohort of schizophrenic subjects	

Isohanni	2014	Outcomes of schizophrenia from a lifespan perspective. The Northern Finland 1966 birth cohort study (NFBC 1966)	
Jalbert	2014	Disease progression, treatment patterns, and outcomes in schizophrenia over a 3-year period: Results from the cohort for the general study of schizophrenia (CGS)	https://dx.doi.org/10.1002/pds.3701
Kanner	2012	Psychiatric comorbidities and epilepsy: Is it the old story of the chicken and the egg?	https://dx.doi.org/10.1002/ana.23679
Karadima	2010	Reasons and patterns of hospitalization among schizophrenic patients in Greece: The Grace study	https://dx.doi.org/10.1186/1744-859X-9-S1-S95
Lagerberg	2022	Reply to Ploderl and Hengartner: Learning about the course of suicidal behavior but not about the effects of SSRIS.	https://dx.doi.org/10.1038/s41386-021-01254-5
Li	2021	Meth use is associated with overdoses, high-risk practices, and adverse social determinants	https://dx.doi.org/10.1097/ADM.0000000000000902
Lindmark	2020	P.303 Treatment outcomes in patients with prescription narcotic use disorder (TAPE)	https://dx.doi.org/10.1016/j.euroneuro.2019.12.066
Lovejoy	2017	Suicidal ideation and behaviors following clinician-initiated prescription opioid discontinuation among long-term opioid users	
Lovrecic	2016	Overdoses and other drug related deaths: Comparison between outpatient treatment registered and not-registered patients	http://www.heroinaddictionrelatedclinicalproblems.org/harcp-archives.php
Manchon	2013	Psychotropics and suicidal behaviour-a case-control study	
Murru	2017	One-year course of illness and clinical management in a cohort of patients affected with schizoaffective and bipolar disorders	
Neligan	2010	Antiepileptic medications and the risk of suicide.	https://dx.doi.org/10.1001/jama.2010.1067

Nieto	2021	Completed suicide in bipolar i patients after their first hospitalisation	https://dx.doi.org/10.1192/j.eurpsy.2021.1656
Ohlund	2021	Self-injurious behaviour in patients with bipolar disorder and attention deficit hyperactivity disorder after central stimulant start- a retrospective study based on the lisie cohort	https://dx.doi.org/10.1192/j.eurpsy.2021.239
Peuskens	2010	Principal outcomes of the Sertindole Cohort Prospective (SCOP) study	https://dx.doi.org/10.1016/j.schres.2010.02.428 PT - Conference Abstract
Pompili	2022	Routine treatment pathways of patients with major depression and active suicidal ideation with intent in Italy: interim results from the ARIANNA observational study	https://dx.doi.org/10.1192/j.eurpsy.2022.397
Reutfors	2017	All-cause mortality in treatment-resistant depression: A register-based cohort study in Sweden	
Reutfors	2015	Suicide risk and side effects from antipsychotics in schizophrenia: A nested case-control study	https://dx.doi.org/10.1002/pds.3838
Rihmer	2015	Pharmacologial prevention of suicide	
Rihmer	2012	Depression, suicide and suicide prevention in Hungary	https://dx.doi.org/10.1016/S0924-9338%2812%2975674-2
Rissanen	2011	Use of antipsychotic medication and suicidality-the Northern Finland Birth Cohort 1966 Study	https://dx.doi.org/10.1097/01.yic.0000399969.31830.3f
Roy	2021	Disease Prevalence, Comorbid Conditions, and Medication Utilization Among Patients with Schizophrenia in the United States.	10.1017/S1092852920002515

Seppala	2017	Treatment-resistant and difficult-to treat schizophrenia as a challenge for clinical practices. Data from Finnish samples: Northern Finland birth cohort 1966 and Perfect-project	https://dx.doi.org/10.1007/s00406-017-0824-8
Stephansson	2010	Prescribed antipsychotic drugs and risk of re-hospitalization among incident patients with schizophrenia-related diagnosis	https://dx.doi.org/10.1002/pds.2019
Strom	2010	The ziprasidone observational study of cardiac outcomes (ZODIAC): Findings from a large simple trial of ziprasidone vs. olanzapine in real-world use among 18154 patients with schizophrenia	https://dx.doi.org/10.1016/j.schres.2010.02.523
Sung	2018	Risk of suicide with concurrent use of benzodiazepine, antidepressant, opioid analgesic, and zolpidem: A population based case-control and case-crossover study	https://dx.doi.org/10.1002/pds.4629
Terao	2018	Mixed features in bipolar I disorder and the effect of lithium on suicide.	https://dx.doi.org/10.1176/appi.ajp.2017.17070759
Tiihonen	2018	What does epidemiological data tell us about clozapine's effectiveness?	https://dx.doi.org/10.1093/schbul/sby014.170
Tiihonen	2018	Response to the editorial on antipsychotics and mortality in a nationwide cohort of 29,823 patients with schizophrenia	https://dx.doi.org/10.1016/j.schres.2018.06.035
Tiihonen	2006	Antidepressant use and the risk of suicide, attempted suicide and overall mortality in a nation-wide cohort	10.1016/S0924-977X(06)70350-9
Valenstein	2006	Antidepressants, Concurrent Treatments, and Completed Suicide in VA Registry Data	
Valuck	2009	General population risk of suicide attempt compared to depressed untreated and antidepressant treated populations	https://dx.doi.org/10.1002/pds.1806

Vieta	2011	Health care resource utilization among patients with bipolar disorder: Retrospective data from a large multinational longitudinal study (WAVE-BD)	
Wen	2009	Is antiepileptic drug use associated with suicidality in patients with epilepsy?	

S4. Funnel plots

Figure S4.1 Funnel plot for risks of suicide mortality in people with schizophrenia spectrum disorders treated with clozapine.

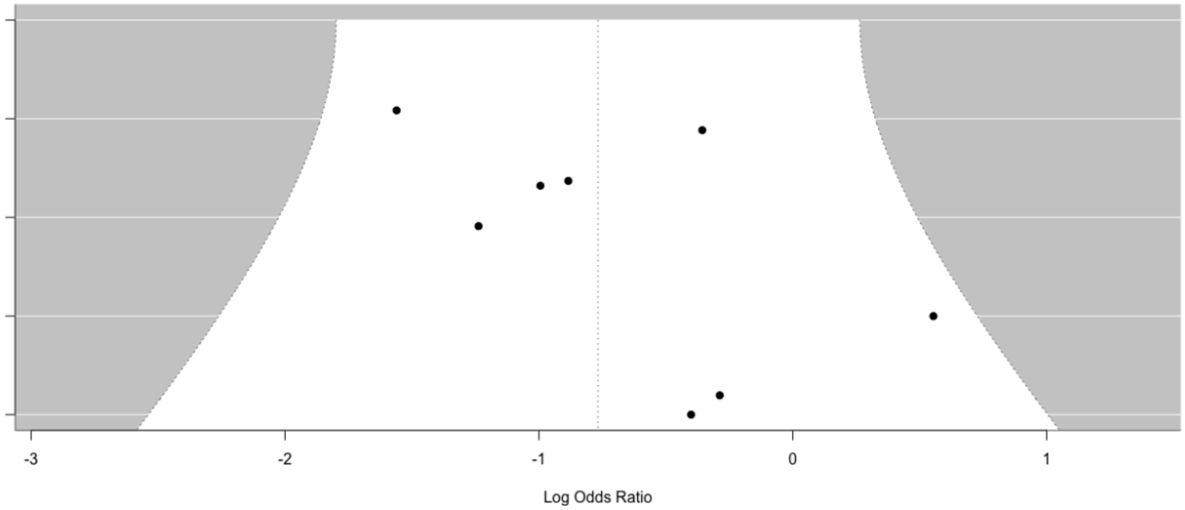

Some visual asymmetry is present, favouring the results with lower risks of suicide mortality.

Figure S4.2 Funnel plot for risks of suicide attempt in people with schizophrenia spectrum disorders treated with clozapine.

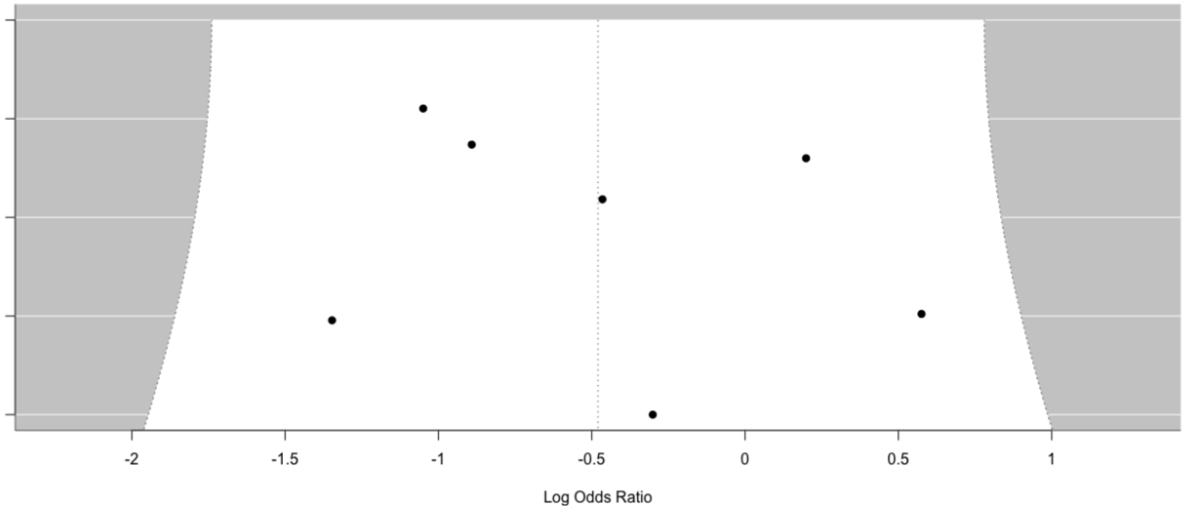

No evidence of visual asymmetry.

Figure S4.3 Funnel plot for risks of suicide mortality in people with bipolar disorder treated with lithium.

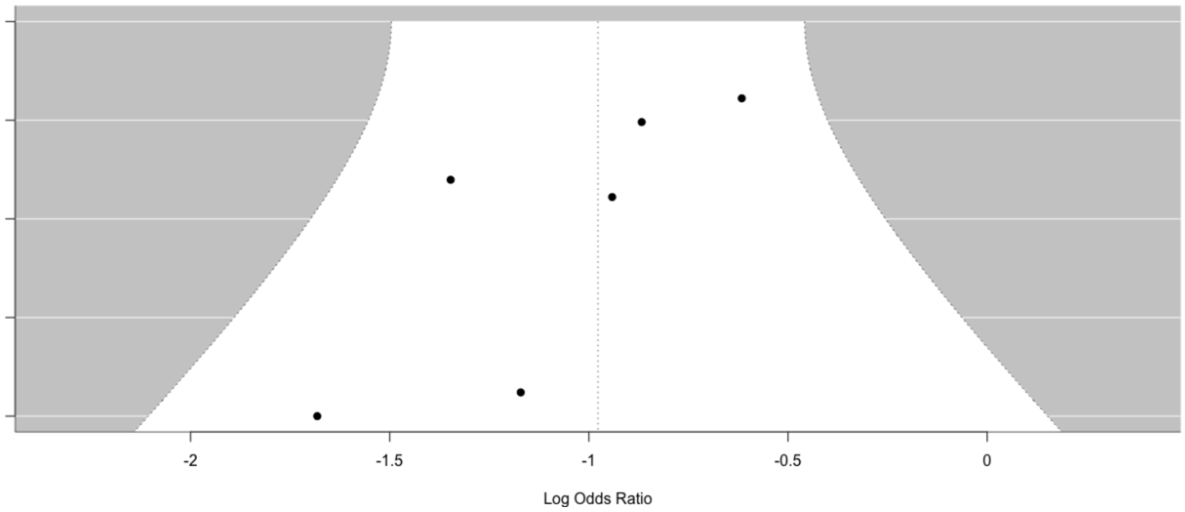

No evidence of visual asymmetry.

Figure S4.4 Funnel plot for risks of suicide attempt in people with bipolar disorder treated with lithium (between-individual studies).

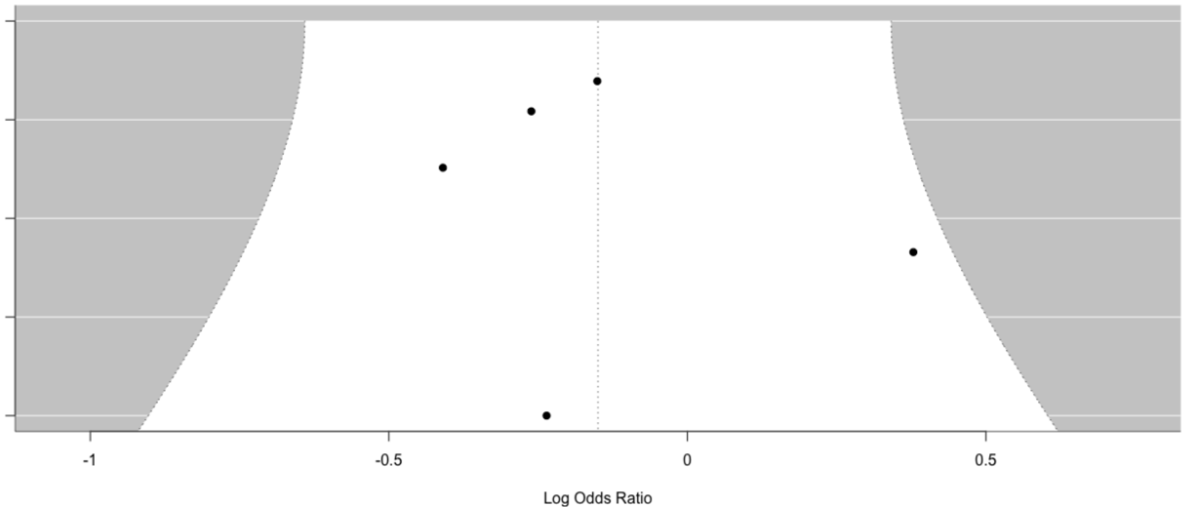

No evidence of visual asymmetry.

Figure S4.4 Funnel plot for risks of suicide attempt in people with bipolar disorder treated with lithium (within-individual studies).

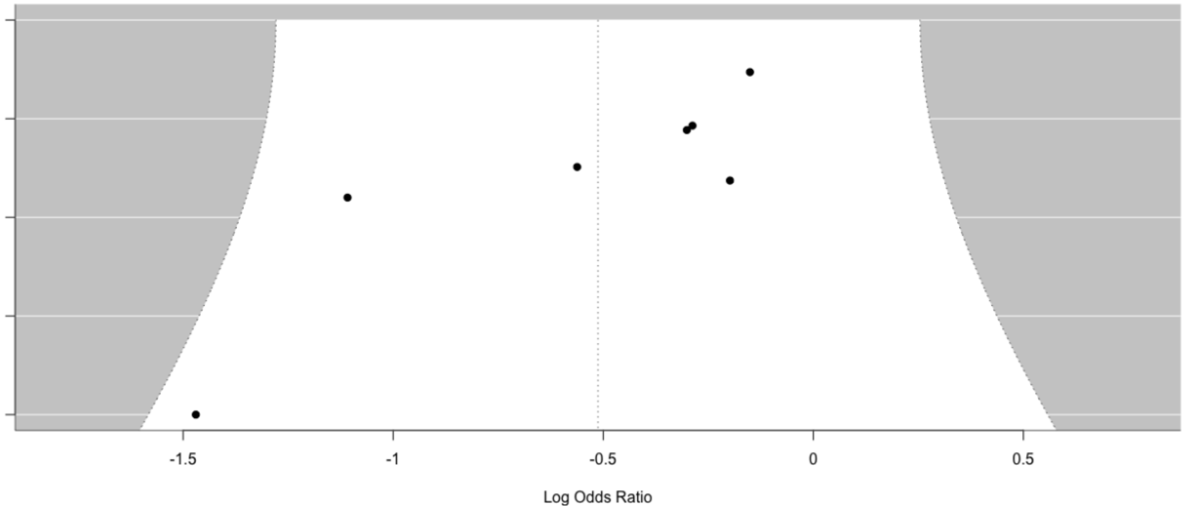

Evidence of visual asymmetry – small imprecise studies show results with lower risks of suicide attempts.

S5. R code for the figures

5.1 Schizophrenia spectrum disorders plot

```
library(ggplot2)

drugs <- c("Antidepressants (unspecified) [2 / ]", "Antipsychotics (unspecified) [2 / 2]",
"First-generation atipsychotics (unspecified) [ / 2]", "SSRIs (unspecified) [ / 4]", "Fluoxetine
[ / 2]", "Aripiprazole [ / 3]", "Flupentixol [2 /3]", "Olanzapine [ / 5]", "Perphenazine [2 / ]",
"Quetiapine [ / 4]", "Zuclopenthixol [3 / 3]", "Benzodiazepines [ / 3]")

wi <- c(1.40, 0.90, NA, NA, NA, NA, 0.73, NA, 0.84, NA, 0.98, NA)
bi <- c(NA, 0.95, 2.16, 0.59, 0.49, 0.61, 0.66, 0.53, NA, 0.75, 0.44, 2.93)

lowerCIwi <- c(1.19, 0.81, NA, NA, NA, NA, 0.53, NA, 0.69, NA, 0.84, NA)
upperCIwi <- c(1.66, 0.99, NA, NA, NA, NA, 1.01, NA, 1.01, NA, 1.16, NA)
labelwi <- c(("1.40;1.19-1.66"),("0.90;0.81-0.99"),NA, NA, NA, NA, ("0.73;0.53-1.01"),
NA, ("0.84;0.69-1.01"), NA, ("0.98;0.84-1.16"), NA)

lowerCIbi <- c(NA, 0.65, 1.39, 0.38, 0.18, 0.37, 0.35, 0.39, NA, 0.58, 0.30, 1.57)
upperCIbi <- c(NA, 1.37, 3.35, 0.89, 1.31, 1.01, 1.25, 0.71, NA, 0.96, 0.63, 5.47)
labelbi <- c(NA, ("0.95;0.65-1.37"),("2.16;1.39-3.35"), ("0.59;0.38-0.89"), ("0.49;0.18-
1.31"), ("0.61;0.37-1.01"), ("0.66;0.35-1.25"),("0.53;0.39-0.71"), NA, ("0.75;0.58-0.96"),
("0.44;0.30-0.63"), ("2.93;1.57-5.47"))


drugs <- factor(drugs, levels = rev(drugs))


data <- data.frame(
  drugs = drugs,
  wi = rev(wi),
  bi = rev(bi),
  lowerCIwi = rev(lowerCIwi),
  upperCIwi = rev(upperCIwi),
  lowerCIbi = rev(lowerCIbi),
  upperCIbi = rev(upperCIbi),
  labelwi = rev(labelwi),
  labelbi = rev(labelbi)
)


data$y_wi <- seq_along(data$drugs)
data$y_bi <- data$y_wi - 0.15

library(ggplot2)
library(dplyr)


xmin <- 0.25
```

```
xmax <- 5
pad  <- 1.05

data <- data %>%
  mutate(
    x_wi_lab = case_when(
      is.na(wi) ~ NA_real_,
      wi < xmin ~ xmin * pad,
      wi > xmax ~ xmax / pad,
      TRUE ~ wi
    ),
    x_bi_lab = case_when(
      is.na(bi) ~ NA_real_,
      bi < xmin ~ xmin * pad,
      bi > xmax ~ xmax / pad,
      TRUE ~ bi
    ),

    hjust_wi = ifelse(!is.na(wi) & wi < xmin, 0, ifelse(!is.na(wi) & wi > xmax, 1, 0.5)),
    hjust_bi = ifelse(!is.na(bi) & bi < xmin, 0, ifelse(!is.na(bi) & bi > xmax, 1, 0.5))
  )

forest_plot <- ggplot(data) +
  geom_point(aes(x = wi, y = y_wi, fill = "Suicide attempts"),
             shape = 21, size = 2.5, colour = "black") +
  geom_point(aes(x = bi, y = y_bi, fill = "Suicide mortality"),
             shape = 21, size = 2.5, colour = "black") +


  geom_text(aes(x = x_wi_lab, y = y_wi, label = labelwi, hjust = hjust_wi),
            vjust = -0.9, size = 2.5, colour = "black") +
  geom_text(aes(x = x_bi_lab, y = y_bi, label = labelbi, hjust = hjust_bi),
            vjust = 1.75, size = 2.5, colour = "black", fontface = "italic") +

  geom_errorbarh(aes(xmin = lowerCIwi, xmax = upperCIwi, y = y_wi),
                 height = 0.2, colour = "lightgrey", size = 0.8) +
  geom_errorbarh(aes(xmin = lowerCIbi, xmax = upperCIbi, y = y_bi),
                 height = 0.2, colour = "darkgrey", size = 0.8) +
  geom_vline(xintercept = 1, linetype = "dashed", colour = "blue") +

  labs(x = "Odds Ratio", y = "") +
  scale_x_log10(breaks = c(0.25, 0.5, 0.75, 1, 2, 3, 5),
                labels = c("0.25","0.50","0.75","1.00","2.00","3.00","5.00")) +
  coord_cartesian(xlim = c(xmin, xmax)) +
  scale_fill_manual(name = "Outcome",
                    values = c("Suicide attempts" = "white",
                               "Suicide mortality" = "darkgrey")) +
  scale_y_continuous(breaks = data$y_wi, labels = levels(data$drugs)) +
```

```
  annotate("segment", x = 4.25, xend = 5.35,
           y = data$y_wi[data$upperCIwi > 5.35],
           yend = data$y_wi[data$upperCIwi > 5.35],
           arrow = arrow(type = "closed", length = unit(0.1, "inches")), colour = "lightgrey") +
  annotate("segment", x = 4.25, xend = 5.35,
           y = data$y_bi[data$upperCIbi > 5.35],
           yend = data$y_bi[data$upperCIbi > 5.35],
           arrow = arrow(type = "closed", length = unit(0.1, "inches")), colour = "darkgray") +
  annotate("segment", x = 0.225, xend = 0.215,
           y = data$y_wi[data$lowerCIwi < 0.22],
           yend = data$y_wi[data$lowerCIwi < 0.22],
           arrow = arrow(type = "closed", length = unit(0.1, "inches")), colour = "lightgrey") +
  annotate("segment", x = 0.225, xend = 0.215,
           y = data$y_bi[data$lowerCIbi < 0.22],
           yend = data$y_bi[data$lowerCIbi < 0.22],
           arrow = arrow(type = "closed", length = unit(0.1, "inches")), colour = "darkgray") +

  theme_minimal() +
  theme(axis.text.y = element_text(size = 9),
        axis.text.x = element_text(size = 9),
        plot.title = element_text(hjust = 0.5, size = 14, face = "bold"),
        legend.position = "right",
        plot.margin = margin(t = 10, r = 10, b = 5, l = 10)) +
  ggtitle("Schizophrenia spectrum disorders")

forest_plot
# Y coord cartesian , ylim = c(min(data$y_bi) + 1.25, max(data$y_wi) - 1)
```

5.2 Bipolar disorder plot (R code)

```
library(ggplot2)

drugs <- c("Antipsychotics (unspecified) [2 / 2]", "Lamotrigine [2 / ]", "Sodium Valproate [4
/ ]", "Valproic acid [ / 3]", "Bezodiazepines [ / 2]")

wi <- c(1.54, 0.86, 1.16, NA, NA)
bi <- c(1.13, NA, NA, 0.66, 3.03)

lowerCIwi <- c(1.36, 0.65, 1.03, NA, NA)
upperCIwi <- c(1.74, 1.14, 1.31, NA, NA)
labelwi <- c(("1.53; 1.36-1.14"), ("0.86; 0.65-1.14"), ("1.16; 1.03-1.31"), NA, NA)

lowerCIbi <- c(0.76, NA, NA, 0.59, 1.72)
upperCIbi <- c(1.68, NA, NA, 0.75, 5.34)
labelbi <- c(("1.13;0.76-1.68"), NA, NA, ("0.66; 0.59-0.75"), ("3.03; 1.72-5-34"))


drugs <- factor(drugs, levels = rev(drugs))


data <- data.frame(
  drugs = drugs,
  wi = rev(wi),
  bi = rev(bi),
  lowerCIwi = rev(lowerCIwi),
  upperCIwi = rev(upperCIwi),
  lowerCIbi = rev(lowerCIbi),
  upperCIbi = rev(upperCIbi),
  labelwi = rev(labelwi),
  labelbi = rev(labelbi)
)


data$y_wi <- seq_along(data$drugs)
data$y_bi <- data$y_wi - 0.15

library(ggplot2)
library(dplyr)


xmin <- 0.25
xmax <- 5
pad  <- 1.05

data <- data %>%
  mutate(
    x_wi_lab = case_when(
```

```
      is.na(wi) ~ NA_real_,
      wi < xmin ~ xmin * pad,
      wi > xmax ~ xmax / pad,
      TRUE ~ wi
    ),
    x_bi_lab = case_when(
      is.na(bi) ~ NA_real_,
      bi < xmin ~ xmin * pad,
      bi > xmax ~ xmax / pad,
      TRUE ~ bi
    ),

    hjust_wi = ifelse(!is.na(wi) & wi < xmin, 0, ifelse(!is.na(wi) & wi > xmax, 1, 0.5)),
    hjust_bi = ifelse(!is.na(bi) & bi < xmin, 0, ifelse(!is.na(bi) & bi > xmax, 1, 0.5))
  )

forest_plot <- ggplot(data) +
  geom_point(aes(x = wi, y = y_wi, fill = "Suicide attempts"),
             shape = 21, size = 2.5, colour = "black") +
  geom_point(aes(x = bi, y = y_bi, fill = "Suicide mortality"),
             shape = 21, size = 2.5, colour = "black") +


  geom_text(aes(x = x_wi_lab, y = y_wi, label = labelwi, hjust = hjust_wi),
            vjust = -0.9, size = 2.5, colour = "black") +
  geom_text(aes(x = x_bi_lab, y = y_bi, label = labelbi, hjust = hjust_bi),
            vjust = 1.75, size = 2.5, colour = "black", fontface = "italic") +

  geom_errorbarh(aes(xmin = lowerCIwi, xmax = upperCIwi, y = y_wi),
                 height = 0.2, colour = "lightgrey", size = 0.8) +
  geom_errorbarh(aes(xmin = lowerCIbi, xmax = upperCIbi, y = y_bi),
                 height = 0.2, colour = "darkgrey", size = 0.8) +
  geom_vline(xintercept = 1, linetype = "dashed", colour = "blue") +

  labs(x = "Odds Ratio", y = "") +
  scale_x_log10(breaks = c(0.25, 0.5, 0.75, 1, 2, 3, 5),
                labels = c("0.25","0.50","0.75","1.00","2.00","3.00","5.00")) +
  coord_cartesian(xlim = c(xmin, xmax)) +
  scale_fill_manual(name = "Outcome",
                    values = c("Suicide attempts" = "white",
                               "Suicide mortality" = "darkgrey")) +
  scale_y_continuous(breaks = data$y_wi, labels = levels(data$drugs)) +


  annotate("segment", x = 4.25, xend = 5.35,
           y = data$y_wi[data$upperCIwi > 5.35],
           yend = data$y_wi[data$upperCIwi > 5.35],
           arrow = arrow(type = "closed", length = unit(0.1, "inches")), colour = "lightgrey") +
  annotate("segment", x = 4.25, xend = 5.35,
           y = data$y_bi[data$upperCIbi > 5.35],
```

```
          yend = data$y_bi[data$upperCIbi > 5.35],
          arrow = arrow(type = "closed", length = unit(0.1, "inches")), colour = "darkgray") +
  annotate("segment", x = 0.225, xend = 0.215,
          y = data$y_wi[data$lowerCIwi < 0.22],
          yend = data$y_wi[data$lowerCIwi < 0.22],
          arrow = arrow(type = "closed", length = unit(0.1, "inches")), colour = "lightgrey") +
  annotate("segment", x = 0.225, xend = 0.215,
          y = data$y_bi[data$lowerCIbi < 0.22],
          yend = data$y_bi[data$lowerCIbi < 0.22],
          arrow = arrow(type = "closed", length = unit(0.1, "inches")), colour = "darkgray") +

  theme_minimal() +
  theme(axis.text.y = element_text(size = 9),
       axis.text.x = element_text(size = 9),
       plot.title = element_text(hjust = 0.5, size = 14, face = "bold"),
       legend.position = "right",
       plot.margin = margin(t = 10, r = 10, b = 5, l = 10)) +
  ggtitle("Bipolar disorder")

forest_plot
# Y coord cartesian , ylim = c(min(data$y_bi) + 1.25, max(data$y_wi) - 1)
```

5.3 Depression plot

```
library(ggplot2)

drugs <- c("Antidepressants (unspecified) [2 / ]", "SSRIs [4 / 2]", "SNRIs and NaSSA [2 / 2]", "Older antidepressants [ / 2]")

wi <- c(1.02, 0.91, 1.07, NA)
bi <- c(NA, 0.61, 0.71, 0.68)

lowerCIwi <- c(0.81,0.72, 0.82, NA)
upperCIwi <- c(1.28,1.16, 1.40, NA)
labelwi <- c("1.02;0.81-1.28","0.91;0.72-1.16", "1.07;0.82-1.40", NA)

lowerCIbi <- c(NA, 0.47, 0.43, 0.59)
upperCIbi <- c(NA, 0.81, 1.17, 0.78)
labelbi <- c(NA, "0.61;0.47-0.81", "0.71;0.43-1.17", "0.68;0.59-0.78")


drugs <- factor(drugs, levels = rev(drugs))


data <- data.frame(
  drugs = drugs,
  wi = rev(wi),
  bi = rev(bi),
  lowerCIwi = rev(lowerCIwi),
  upperCIwi = rev(upperCIwi),
  lowerCIbi = rev(lowerCIbi),
  upperCIbi = rev(upperCIbi),
  labelwi = rev(labelwi),
  labelbi = rev(labelbi)
)

data$y_wi <- seq_along(data$drugs)
data$y_bi <- data$y_wi - 0.15

library(ggplot2)
library(dplyr)


xmin <- 0.25
xmax <- 5
pad  <- 1.05
data <- data %>%
  mutate(

    x_wi_lab = case_when(
      is.na(wi) ~ NA_real_,
```

```
    wi < xmin ~ xmin * pad,
    wi > xmax ~ xmax / pad,
    TRUE ~ wi
  ),
  x_bi_lab = case_when(
    is.na(bi) ~ NA_real_,
    bi < xmin ~ xmin * pad,
    bi > xmax ~ xmax / pad,
    TRUE ~ bi
  ),

  hjust_wi = ifelse(!is.na(wi) & wi < xmin, 0, ifelse(!is.na(wi) & wi > xmax, 1, 0.5)),
  hjust_bi = ifelse(!is.na(bi) & bi < xmin, 0, ifelse(!is.na(bi) & bi > xmax, 1, 0.5))
 )

forest_plot <- ggplot(data) +
  geom_point(aes(x = wi, y = y_wi, fill = "Suicide attempts"),
             shape = 21, size = 2.5, colour = "black") +
  geom_point(aes(x = bi, y = y_bi, fill = "Suicide mortality"),
             shape = 21, size = 2.5, colour = "black") +

  geom_text(aes(x = x_wi_lab, y = y_wi, label = labelwi, hjust = hjust_wi),
            vjust = -0.9, size = 2.5, colour = "black") +
  geom_text(aes(x = x_bi_lab, y = y_bi, label = labelbi, hjust = hjust_bi),
            vjust = 1.75, size = 2.5, colour = "black", fontface = "italic") +

  geom_errorbarh(aes(xmin = lowerCIwi, xmax = upperCIwi, y = y_wi),
                 height = 0.2, colour = "lightgrey", size = 0.8) +
  geom_errorbarh(aes(xmin = lowerCIbi, xmax = upperCIbi, y = y_bi),
                 height = 0.2, colour = "darkgrey", size = 0.8) +
  geom_vline(xintercept = 1, linetype = "dashed", colour = "blue") +

  labs(x = "Odds Ratio", y = "") +
  scale_x_log10(breaks = c(0.25, 0.5, 0.75, 1, 2, 3, 5),
                labels = c("0.25","0.50","0.75","1.00","2.00","3.00","5.00")) +
  coord_cartesian(xlim = c(xmin, xmax)) +
  scale_fill_manual(name = "Outcome",
                    values = c("Suicide attempts" = "white",
                               "Suicide mortality" = "darkgrey")) +
  scale_y_continuous(breaks = data$y_wi, labels = levels(data$drugs)) +


  annotate("segment", x = 4.25, xend = 5.35,
           y = data$y_wi[data$upperCIwi > 5.35],
           yend = data$y_wi[data$upperCIwi > 5.35],
           arrow = arrow(type = "closed", length = unit(0.1, "inches")), colour = "lightgrey") +
  annotate("segment", x = 4.25, xend = 5.35,
           y = data$y_bi[data$upperCIbi > 5.35],
           yend = data$y_bi[data$upperCIbi > 5.35],
           arrow = arrow(type = "closed", length = unit(0.1, "inches")), colour = "darkgray") +
```

```
  annotate("segment", x = 0.225, xend = 0.215,
           y = data$y_wi[data$lowerCIwi < 0.22],
           yend = data$y_wi[data$lowerCIwi < 0.22],
           arrow = arrow(type = "closed", length = unit(0.1, "inches")), colour = "lightgrey") +
  annotate("segment", x = 0.225, xend = 0.215,
           y = data$y_bi[data$lowerCIbi < 0.22],
           yend = data$y_bi[data$lowerCIbi < 0.22],
           arrow = arrow(type = "closed", length = unit(0.1, "inches")), colour = "darkgray") +

  theme_minimal() +
  theme(axis.text.y = element_text(size = 9),
        axis.text.x = element_text(size = 9),
        plot.title = element_text(hjust = 0.5, size = 14, face = "bold"),
        legend.position = "right",
        plot.margin = margin(t = 10, r = 10, b = 5, l = 10)) +
  ggtitle("Depression")

forest_plot
# Y coord cartesian , ylim = c(min(data$y_bi) + 1.25, max(data$y_wi) - 1)
```
